# Analysis of humoral and cellular immune activation up to 21 months after heterologous and homologous COVID-19 vaccination

**DOI:** 10.3389/fimmu.2025.1579163

**Published:** 2025-05-02

**Authors:** Davide Torre, Chiara Orlandi, Ilaria Conti, Simone Barocci, Eugenio Carlotti, Mauro Magnani, Anna Casabianca, Giuseppe Stefanetti

**Affiliations:** ^1^ Department of Biomolecular Sciences, University of Urbino Carlo Bo, Urbino, Italy; ^2^ Department of Biomolecular Sciences, Section of Biochemistry and Biotechnology, University of Urbino Carlo Bo, Fano, Italy; ^3^ Laboratorio Covid, University of Urbino Carlo Bo, Fano, Italy; ^4^ Department of Clinical Pathology, Azienda Sanitaria Territoriale (AST) di Pesaro-Urbino, Urbino, Italy; ^5^ Department of Prevention, Azienda Sanitaria Territoriale (AST) di Pesaro-Urbino, Urbino, Italy

**Keywords:** SARS-CoV-2, COVID-19, heterologous vaccine, anti-spike IgG response, T-cell response, B-cell response, long-term immunity, hybrid immunity

## Abstract

To address the COVID-19 pandemic, diverse vaccination strategies, including homologous and heterologous schedules, were employed to enhance immune protection. This study evaluates the long-term humoral and cellular immune responses in individuals vaccinated with homologous (ChAdOx1-S/ChAdOx1-S [ChAd/ChAd]) and heterologous (ChAdOx1-S/BNT162b2 [ChAd/BNT]) schedules, followed by a third-dose mRNA booster (BNT162b2 [BNT] or mRNA-1273). Anti-Spike IgG titers were measured at 9-, 12-, and 21-months post-primary vaccination (corresponding to 3-, 6-, and 15-months post-booster), while SARS-CoV-2-specific B- and T-cell responses were assessed at 21-months post-booster. Antibody titers declined by 12-months post-primary vaccination, regardless of the third dose administered, and increased significantly by 21-months, potentially due to a fourth dose (BNT or mRNA-1273) or natural SARS-CoV-2 infection. The heterologous ChAd/BNT schedule elicited a stronger and more durable immune response than the homologous ChAd/ChAd, as evidenced by higher anti-Spike IgG titers, increased IgM-/IgG+ memory B-cell activation, and enhanced cytotoxic CD8+ T-cell cytokine expression in infected individuals. SARS-CoV-2 infection further boosted humoral and cellular responses, with infected individuals showing higher anti-Spike IgG titers and greater CD8+ T-cell activation compared to uninfected individuals. These findings highlight the benefits of heterologous vaccination schedules and the role of infection-driven immune activation, providing valuable insights for optimizing vaccination strategies to improve long-term immunity against SARS-CoV-2.

## 1 Introduction

The coronavirus disease 2019 [COVID-19] pandemic fundamentally transformed both the vaccine development process and global vaccination strategies to combat the rapid spread of Severe Acute Respiratory Syndrome Coronavirus 2 [SARS-CoV-2] and its associated mortality (1). Within less than a year, several vaccine platforms—including mRNA-based vaccines (e.g., Pfizer-BioNTech BNT162b2 [BNT] and Moderna [mRNA-1273]), viral vector vaccines (e.g., Oxford-AstraZeneca ChAdOx1-S nCoV-19 [ChAd]), and protein subunit vaccines (e.g., Novavax Nuvavoxid [NVX-CoV2373]) —were developed and received emergency use authorization, an unprecedented achievement in medical science (2). To address increasing public health concerns and supply chain constraints, novel vaccination strategies were adopted, based on current available vaccines. While many individuals received homologous vaccination schedules—using the same vaccine for both doses—others received heterologous schedules, combining different vaccine platforms (3, 4). Notably, heterologous vaccination has also been employed for booster doses, demonstrating improved neutralizing antibody titers and enhanced T-cell responses compared to homologous regimens (5). Despite these promising outcomes, the effectiveness and safety of different vaccine strategies continue to be closely monitored in real-time to evaluate immune response durability and identify adverse effects, such as vaccine-induced thrombosis with thrombocytopenia [VITT], which briefly halted the use of adenoviral vector vaccines like Janssen’s COVID-19 vaccine (6, 7).

Previously, we conducted both cross-sectional and longitudinal analyses of humoral responses from a voluntary cohort in the northern Marche region of Italy (8, 9). In these studies, antibody levels against the SARS-CoV-2 spike [S] protein were compared among individuals who received homologous adenoviral-vector (ChAd/ChAd) or mRNA-based (BNT/BNT) vaccinations and those who received heterologous ChAd/BNT vaccinations. At two months post-primary vaccination, heterologous ChAd/BNT schedules elicited significantly higher anti-spike IgG titers than either homologous schedule (8). Follow-up analyses at four and six months confirmed the robustness of the immune response induced by heterologous schedules, in terms of both higher level and longer-lasting anti-S IgG response; despite a decline in IgG titers over time, which eventually resulted in comparable antibody levels between ChAd/BNT and BNT/BNT groups. Finally, focusing on clinical variables such as sex, age, smoking and body mass index, we observed that only the vaccine schedule influenced anti-S IgG titers at all time points (9).

Building on this foundation, the current study shifts focus on the impact of third dose (booster) mRNA vaccinations and long-term immunity. Approximately six months after primary immunization, participants received a booster dose (BNT or mRNA-1273). This study evaluates the long-term humoral and cellular responses following booster administration, measuring anti-S IgG levels at 9-, 12-, and 21-months post-primary vaccination (corresponding to 3-, 6-, and 15-months post-booster). Cellular immune responses, including T- and B-cell activation, were assessed at 21-months post-primary vaccination (15 months post-booster) to understand the role of vaccination schedules and infection-driven immune activation. The study also investigates the impact of natural SARS-CoV-2 infection on immune system activation. These analyses aim to provide a comprehensive understanding of how different vaccination strategies shape both humoral and cellular immunity over an extended period and contribute to the growing body of evidence informing optimal COVID-19 vaccination strategies.

## 2 Materials and methods

### 2.1 Recruitment and study cohort characteristics

The study participants (n = 203) were recruited among personnel from the University of Urbino, Carlo Bo (Urbino (PU), Italy), vaccinated against COVID-19 between December 2020 and June 2021, and that subsequently received a third (booster) dose administration (BNT162b2 or mRNA-1273) between October 2021 and January 2022. These individuals were a subset of a larger cohort previously studied for their humoral response to COVID-19 vaccination (8, 9). Follow up evaluations were conducted at 3 months (n = 195), 6 months (n = 173) and 15 months (n = 99) after booster immunization. These time points corresponded to approximately 9, 12 and 21 months after the primary immunizations (**Table 1**, **Supplementary Table S1**, **Supplementary Figure S1**). Overall, 87 individuals completed assessments at all three time points (9, 12 and 21 months after the primary immunizations), 90 participants attended two time points, and 26 individuals provided samples at a single time point (**Supplementary Figure S2**, **Supplementary Table S2**). In addition, a small subset of the cohort (20/203), between August 2021 and January 2023, also received a fourth vaccine dose, with differences observed based on vaccination schedules (**Supplementary Table S3**). Serological samples were obtained at each time point for humoral response analysis (**Table 1A**), while cellular response analyses (antigen-specific B- and T-cells) were conducted using samples collected at 21 months after the primary immunizations (**Table 1B**).

**Table 1:** Demographic characteristics of participants in humoral and cellular analyses.

A. Subjects involved for humoral analysis						
Vaccine schedule				9 months	12 months	21 months
Primary	Booster					
All	Total	Gender Age BMI	Male Years, median (IQR) Median (IQR)	*n* = 195 88/195 (45.1%) 55.0 (26.0-72.0) 24.1 (16.2-37.6)	*n* = 173 74/173 (42.8%) 55 (31-72) 24.1 (37.6-16.2)	*n* = 99 39/99 (39.4%) 56.0 (32.0-72.0) 24.2 (16.2-37.3)
	mRNA-1273	Gender Age BMI	Male Years, median (IQR) Median (IQR)	*n* = 127 (65.1%) 59/127 (46.5%) 55.0 (26.0-71.0) 24.1 (16.2-37.6)	*n* = 116 (67.1%) 51/116 (44.0%) 55.0 (31.0-70.0) 24.0 (16.2-37.6)	*n* = 62 (62.6%) 26/62 (41.9%) 57.0 (32.0-69.0) 24.4 (16.2-37.3)
	BNT	Gender Age BMI	Male Years, median (IQR) Median (IQR)	*n* = 68 (34.9%) 29/68 (42.6%) 53.0 (32.0-72.0) 24.2 (19.0-36.7)	*n* =57 (32.9%) 23/57 (40.4%) 54.0 (32.0-72.0) 24.4 (19.0-36.7)	*n* = 37 (37.4%) 13/37 (35.1%) 55.0 (32.0-72.0) 24.0 (19.0-36.7)
ChAd/ChAd	Total	Gender Age BMI	Male Years, median (IQR) Median (IQR)	*n* = 134 (68.7%) 88/134 (65.7%) 55.0 (30.0-72.0) 24.0 (16.2-37.6)	*n* = 124 (71.7%) 57/124 (46.0%) 55.0 (31.0-72.0) 24.1 (16.2-37.6)	*n* = 69 (69.7%) 29/69 (42.0%) 57.0 (32.0-72.0) 24.5 (16.2-37.3)
	mRNA-1273	Gender Age BMI	Male Years, median (IQR) Median (IQR)	*n* = 111 (65.1%) 52/111 (46.8%) 56.0 (30.0-71.0) 24.0 (16.2-37.6)	*n* = 105 (67.1%) 48/105 (45.7%) 55.0 (31.0-70.0) 23.8 (16.2-37.6)	*n* = 56 (62.6%) 24/56 (42.9%) 57.0 (32.0-69.0) 24.4 (16.2-37.3)
	BNT	Gender Age BMI	Male Years, median (IQR) Median (IQR)	*n* = 23 (34.9%) 9/23 (39.1%) 55.0 (32.0-72.0) 24.5 (19.5-36.2)	*n* = 19 (32.9%) 9/19 (47.4%) 55.0 (37.0-72.0) 25.1 (20.5-36.2)	*n* = 13 (37.4%) 5/13 (38.5%) 60.0 (36.0-72.0) 25.3 (19.5-36.2)
ChAd/BNT	Total	Gender Age BMI	Male Years, median (IQR) Median (IQR)	*n* = 61 (31.3%) 27/61 (44.3%) 53.0 (26.0-62.0) 24.3 (18.7-36.7)	*n* = 49 (28.3%) 17/49 (34.7%) 54.0 (32.0-61.0) 24.1 (18.7-36.7)	*n* = 30 (30.3%) 10/30 (33.3%) 54.5 (32.0-61.0) 23.4 (18.7-36.7)
	mRNA-1273	Gender Age BMI	Male Years, median (IQR) Median (IQR)	*n* = 16 (65.1%) 7/16 (43.8%) 54.5 (26.0-61.0) 25.5 (18.7-35.6)	*n* = 11 (67.1%) 3/11 (27.3%) 55.0 (45.0-61.0) 25.4 (18.7-35.6)	*n* = 6 (62.6%) 2/6 (33.3%) 56.5 (45.0-61.0) 23.8 (18.7-33.5)
	BNT	Gender Age BMI	Male Years, median (IQR) Median (IQR)	*n* = 45 (34.9%) 20/45 (44.4%) 52.0 (32.0-62.0) 24.0 (19.0-36.7)	*n* = 38 (32.9%) 14/38 (36.8%) 53.0 (32.0-60.0) 23.8 (19.0-36.7)	*n* = 24 (37.4%) 8/24 (33.3%) 53.5 (32.0-60.0) 23.4 (19.0-36.7)

B. Subjects involved for cellular analysis					
Vaccine schedule				B-cells	T-cells
Primary	Booster				
All	Total	N. of individuals (% on total)		*n* = 23 (23.2%)	*n* =24 (24.2%)
		Gender	Male	10/23 (43.5%)	11/24 (45.8%)
		Age	Years, median (IQR)	55.0 (32.0-67.0)	54.5 (32.0-67.0)
		BMI	Median (IQR)	24.0 (18.1-30.2)	23.8 (18.1-30.2)
	mRNA-1273	N. of individuals (% on total)		*n* = 10 (10.1%)	*n* = 11 (11.1%)
		Gender	Male	5/10 (50.0%)	6/11 (54.5%)
		Age	Years, median (IQR)	54.5 (32.0-67.0)	54.0 (32.0-67.0)
		BMI	Median (IQR)	25.1 (18.1-30.2)	24.8 (18.1-30.2)
	BNT	N. of individuals (% on total)		*n* = 12 (12.1%)	*n* = 12 (12.1%)
		Gender	Male	4/12 (33.3%)	4/12 (33.3%)
		Age	Years, median (IQR)	55.5 (36.0-66.6)	55.5 (36.0-66.6)
		BMI	Median (IQR)	23.6 (19.0-28.3)	23.6 (19.0-28.3)
ChAd/ChAd	Total	N. of individuals (% on total)		*n* = 12 (12.1%)	*n* = 12 (12.1%)
		Gender	Male	7/12 (58.3%)	7/12 (58.3%)
		Age	Years, median (IQR)	53.0 (32.0-67.0)	53.0 (32.0-67.0)
		BMI	Median (IQR)	24.3 (18.1-30.2)	24.3 (18.1-30.2)
	mRNA-1273	N. of individuals (% on total)		*n* = 7 (7.1%)	*n* = 7 (7.1%)
		Gender	Male	4/7 (57.1%)	4/7 (57.1%)
		Age	Years, median (IQR)	52.0 (32.0-67.0)	52.0 (32.0-67.0)
		BMI	Median (IQR)	26.3 (18.1-30.2)	26.3 (18.1-30.2)
	BNT	N. of individuals (% on total)		*n* = 4 (4.0%)	*n* = 4 (4.0%)
		Gender	Male	2/4 (50.0%)	2/4 (50.0%)
		Age	Years, median (IQR)	58.0 (36.0-66.0)	58.0 (36.0-66.0)
		BMI	Median (IQR)	22.3 (19.5-24.5)	22.3 (19.5-24.5)
ChAd/BNT	Total	N. of individuals (% on total)		*n* = 11 (11.1%)	*n* = 12 (12.1%)
		Gender	Male	3/11 (27.3%)	4/12 (33.3%)
		Age	Years, median (IQR)	55.0 (51.0-58.0)	55.0 (51.0-58.0)
		BMI	Median (IQR)	23.1 (18.7-28.3)	22.7 (18.7-28.3)
	mRNA-1273	N. of individuals (% on total)		*n* = 3 (3.0%)	*n* = 4 (4.0%)
		Gender	Male	1/3 (33.3%)	2/4 (50.0%)
		Age	Years, median (IQR)	58.0 (55.0-58.0)	56.5 (52.0-58.0)
		BMI	Median (IQR)	20.2 (18.7-25.4)	21.2 (20.2-25.4)
	BNT	N. of individuals (% on total)		*n* = 8 (8.1%)	*n* = 8 (8.1%)
		Gender	Male	2/8 (25.0%)	2/8 (25.0%)
		Age	Years, median (IQR)	54.5 (51.0-58.0)	54.5 (51.0-58.0)
		BMI	Median (IQR)	24.2 (19.0-28.3)	24.2 (19.0-28.3)

ChAd/ChAd refers to the ChAdOx1 COVID-19 vaccine [ChAd] administered as both the first and second doses (primary immunization), while ChAd/BNT refers to ChAd as the first dose and the BNT162b2 COVID-19 vaccine [BNT] as the second dose (primary immunization), IQR: interquartile range (25^th^-75^th^ IQR within brackets).

Additional subgrouping included participants with a confirmed SARS-CoV-2 infection during the study period (**Supplementary Figure S3**). Participants were classified as N+ (nucleocapsid-positive) if they tested positive for SARS-CoV-2 nucleocapsid-specific IgG and/or IgM antibodies, or N-(nucleocapsid-negative). Infection rates increased significantly from 9 to 21 months, with variations observed based both on vaccination schedules and booster types, particularly at both 12 and 21 months, where the group ChAd/BNT/mRNA-1273 showed the highest percentage of infected individuals (63.6% and 83.3%, respectively) (**Supplementary Figure S3**, **Supplementary Table S4**). None of the participants declared reinfection during the study period.

### 2.2 Blood collection and serum separation and mononuclear cell isolation

Whole blood samples were collected at the Laboratory of Clinical Pathology (with certified quality management UNI EN ISO 9001:2015) of Urbino Hospital (AST Azienda Sanitaria Territoriale Pesaro - Urbino) using serum separator tubes (SST) with a gel barrier. The samples were allowed to clot at room temperature for 30 minutes before being centrifuged at 1,500 × g for 10 minutes. This procedure yielded clear serum, separated from blood cells by the gel barrier. Serum was then aliquoted within a few hours and stored at -80°C until further analysis. To avoid potential alterations of immunological readouts, no heat treatment of serum samples was performed.

For PBMC isolation, blood samples collected in EDTA vacuettes were processed within 96 hours of collection. PBMCs were separated using Lymphoprep™ density gradient medium. following a standard density gradient centrifugation protocol. The isolated PBMCs were cultured overnight at 37°C in humified atmosphere containing 5% CO_2_ in Roswell Park Memorial Institute 1640 [RPMI-1640] complete medium supplemented with 25 mM HEPES, 2 mM L-glutamine. 1% penicillin-streptomycin and 10% fetal bovine serum [FBS]. Alternatively, PBMCs were cryopreserved at -80°C in FBS supplemented with 10% dimethyl sulfoxide [DMSO] for subsequent analyses.

### 2.3 Determination of antibody levels

Serum samples were analyzed for SARS-CoV-2 IgG antibodies using the “LIAISON^®^ SARS-CoV-2 TrimericS IgG” Chemiluminescent Immunoassay (CLIA) kit, as previously described (8), at the Clinical Pathology Laboratory of the Urbino Hospital (AST Azienda Sanitaria Territoriale Pesaro – Urbino). Serum storage prior to testing was limited to less than 4 days to maintain sample integrity. The assay has high sensitivity (98.7%) and specificity (99.5%) for detecting anti-trimeric SARS-CoV-2 Spike protein IgG antibodies.

The method demonstrates a strong positive percent agreement (95% CI: 97.8–100.0%) and a negative percent agreement of 96.9% (95% CI: 92.9–98.7%) when compared with neutralizing IgG antibodies. The quantification range is 4.81–2080 BAU/mL, with a cut-off value of 33.8 BAU/mL for positivity. Results were expressed in binding antibody units per milliliter (BAU/mL) using a conversion factor of 2.6 (1 BAU/mL = 2.6 AU/mL) (10, 11).

For samples with IgG titers exceeding 2080 BAU/mL, the LIAISON^®^ TrimericS IgG Diluent Accessory was used for dilution according to the manufacturer’s recommendations (1:20 or 1:5 dilution factor, as appropriate) before re-testing to ensure accurate quantification. All the serum samples were assayed for the nucleocapsid-specific IgM and/or IgG antibodies (COVID-19 ELISA IgM and COVID-19 ELISA IgG kits, Diatheva srl, Cartoceto, PU, Italy), following the manufacturer’s instructions, and were classified as N+-(nucleocapsid-positive) or N-(nucleocapsid-negative) based on the presence or absence of SARS-CoV-2 nucleocapsid-specific IgG and/or IgM antibodies.

### 2.4 Activation marker and intracellular cytokine assays

The analysis of SARS-CoV-2-specific T cell responses was performed using the SARS-CoV-2 Prot_S T Cell Analysis Kit (Miltenyi Biotec). Approximately 1 × 10⁶ thawed or freshly isolated PBMCs were rested overnight at 37°C. Subsequently, the PBMCs were cultured in 96-well plates for 6 hours in the presence of 15-mer peptides with an 11-amino-acid overlap spanning the complete coding sequence (amino acids 5–1273) of the SARS-CoV-2 spike (S) glycoprotein (GenBank MN908947.3, Protein QHD43416.1), as provided in the PepTivator^®^ SARS-CoV-2 Prot_S Complete, premium grade (Miltenyi Biotech). After 2 hours of stimulation, Brefeldin A was added at a final concentration of 1 μg/mL to block cytokine secretion.

Following stimulation, cells were washed with phosphate buffer supplemented with 2 mM EDTA and 0.5% bovine serum albumin (BSA) [PEB buffer]. The cells were then fixed and permeabilized before staining for 20 minutes with an antibody mix containing the following markers: CD3-APC, CD4-Vio^®^ Bright B515, CD8-VioGreen™, IFN-γ-PE, TNF-α-PE-Vio770, CD14-VioBlue^®^, CD20-VioBlue^®^, and CD154-APC-Vio770, Viability 405/452 Fixable Dye was used to identify and exclude dead cells.

Sample acquisition was performed on a BD FACS Canto II flow cytometer. The gating strategy to identify activation markers and intracellular cytokine production in CD4⁺ and CD8⁺ T cell populations was as follows: Lymphocyte gate > DEAD/CD14-/CD20- > CD3+ > CD4+ or CD8+ > mean fluorescence intensity [MFI]of TNF-α, IFN-γ, or CD154, and percentage of positive cells (**Supplementary Figure S4A**).

### 2.5 Quantification of SARS-CoV-2 specific B-cells

The analysis of SARS-CoV-2-specific B-cells was performed using the SARS-CoV-2 Spike B Cell Analysis Kit, anti-human (Miltenyi Biotec). Approximately 5–10 × 10⁶ thawed PBMCs were rested overnight at 37°C in complete RPMI-1640 medium. Following incubation, PBMCs were washed in PEB buffer and stained with an antibody cocktail containing the following reagents: Recombinant SARS-CoV-2 Spike-Protein (HEK)-Biotin-Streptavidin-PE, Recombinant SARS-CoV-2 Spike-Protein (HEK)-Biotin-Streptavidin-PE-Vio^®^ 770, CD19-APC-Vio^®^ 770, CD27-Vio Bright FITC. IgG-VioBlue^®^ and IgM-APC. Live/dead cell discrimination was performed using 7-AAD staining.

Samples were acquired on a BD FACS Canto II flow cytometer. The gating strategy to identify SARS-CoV-2-specific memory B-cells was as follows: Lymphocyte gate > Single cells > Live/Dead- > CD19+ > CD27+ > IgG+/IgM- or IgG-/IgM+ > Spike-Protein-PE+/PE-Vio^®^ 770+ (**Supplementary Figure S4B**).

### 2.6 Statistical analysis

To compare IgG levels between two independent vaccination groups, the Mann-Whitney U test was employed. For comparisons of IgG levels involving three or more vaccination groups, or for repeated measures within the same group across different time points, the Kruskal-Wallis test followed by Dunn’s multiple comparisons post-test was applied.

The relationship between vaccination groups and clinical or demographic variables was assessed using the chi-square test for categorical variables and the Kruskal-Wallis test followed by Dunn’s multiple comparisons post-test for continuous variables.

For the analysis of cellular responses, the Kruskal-Wallis test followed by Dunn’s multiple comparisons post-test was used for comparisons involving three or more groups. The Mann-Whitney U test was used for comparisons between two independent groups.

A p-value of less than 0.05 was considered statistically significant. All analyses and visualizations, including box-and-whisker plots, were performed using GraphPad Prism software (version 8.4.2; GraphPad Software, San Diego, CA, USA). The specific statistical test used for each comparison is indicated in the legends.

## 3 Results

### 3.1 Longitudinal analysis of SARS-CoV-2 anti-trimeric Spike IgG levels at 9-, 12- and 21-months post-vaccination

**Figure 1:**
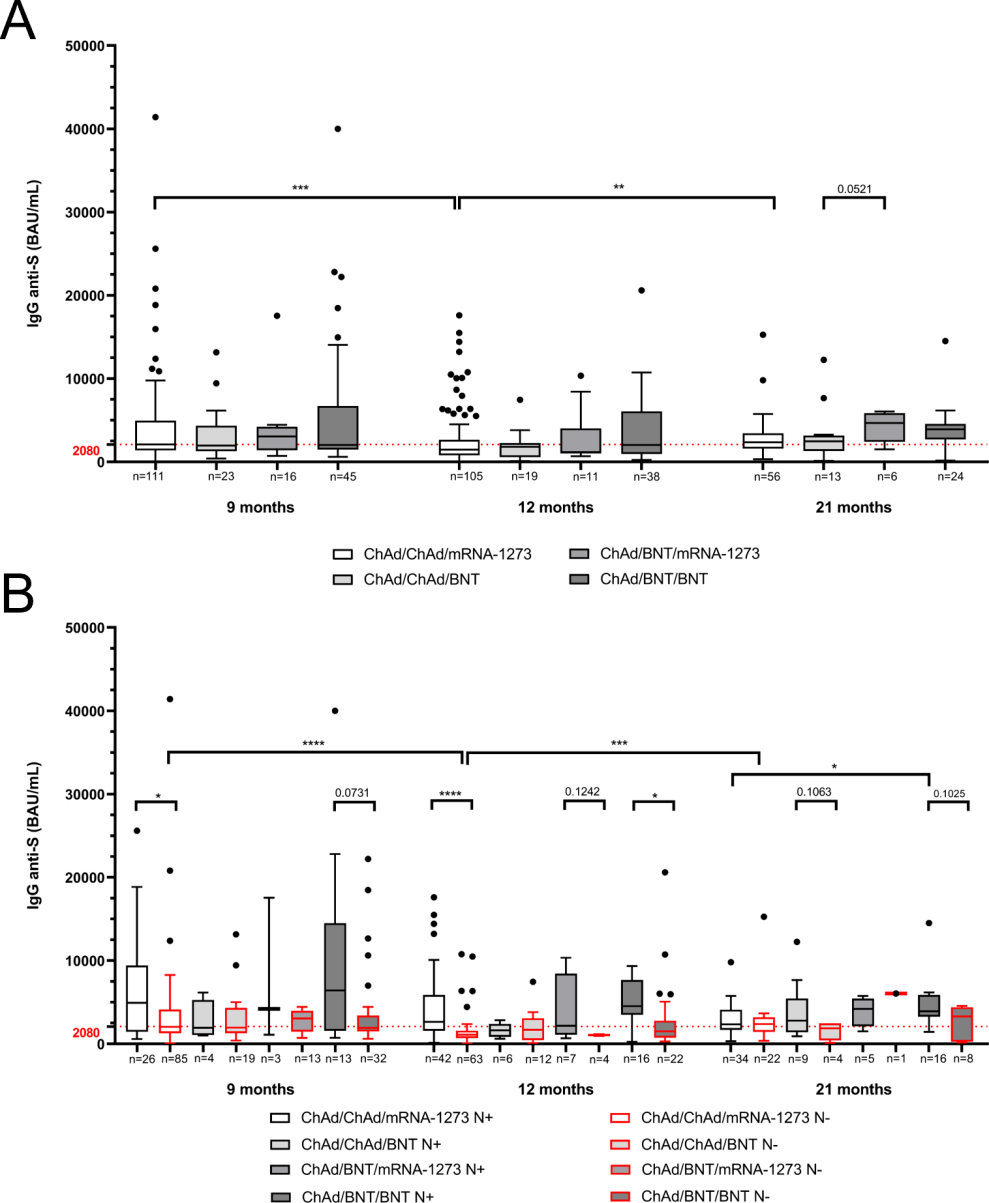
Inter-group comparison of SARS-CoV-2 anti-trimeric Spike protein IgG levels (BAU/mL) among the four vaccination groups at 9, 12 and 21 months post-primary vaccination. **(A)** IgG levels across all vaccinated subjects. **(B)** Comparison of IgG titers between anti-Nucleocapsid-positive [N+] and anti-Nucleocapsid-negative [N−] subjects within each vaccination group. Boxplots show the median and interquartile range [IQR], with whiskers representing the lowest and highest values (Tukey-style). Outliers are displayed as individual points. Statistical comparisons were performed using the Kruskal-Wallis test with Dunn’s *post hoc* multiple comparisons and the Mann-Whitney U test for pairwise comparisons. *p < 0.05; **p < 0.01; ***p < 0.001; ****p < 0.0001. Results for the ChAd/BNT/mRNA-1273 N− group at 21 months are not shown due to the presence of only a single subject, precluding meaningful statistical comparisons.

A positive SARS-CoV-2 anti-trimeric Spike IgG antibody response was observed across all vaccination groups throughout the study period. The longitudinal analysis of antibody titers at 9, 12, and 21 months post-primary vaccination revealed significant temporal variations, particularly when comparing homologous and heterologous vaccination strategies. In the ChAd/ChAd/mRNA-1273 group, a significant decrease in IgG titers was observed from month 9 to 12 (*p* < 0.001), followed by a statistically significant increase between months 12 and 21 (*p* < 0.01) (**Table 2**, **Figure 1A**). In contrast, participants in the ChAd/ChAd/BNT, ChAd/BNT/mRNA-1273 and ChAd/BNT/BNT groups showed a similar pattern of antibody decline at month 12 followed by recovery at month 21; however, these changes were not statistically significant.

**Table 2:** SARS-CoV-2 anti-trimeric Spike IgG titers by vaccination schedule and time points.

IgG titer (BAU/mL)	ChAd/ChAd/ mRNA-1273	ChAd/ChAd/ BNT	ChAd/BNT/ mRNA-1273	ChAd/BNT/ BNT
9 months Median (IQR)	n = 111 (56.9%) 2100 (39.3-41400)	n = 23 (11.8%) 1944 (395-13160)	n = 16 (8.2%) 3070 (711-17540)	n = 45 (23.1%) 2020 (605-40000)
12 months Median (IQR)	n = 105 (60.7%) 1470 (39.2-17600)	n = 19 (11%) 1800 (102-7460)	n = 11 (6.3%) 1148 (670-10340)	n = 38 (22.0%) 2035 (237-20600)
21 months Median (IQR)	n = 56 (56.6%) 2332.5 (333-15260)	n = 13 (13.1%) 2480 (117-12240)	n = 6 (6.1%) 4647.5 (1515-6050)	n = 24 (24.2%) 3915 (165-14500)

Median and interquartile range [IQR] of SARS-CoV-2 anti-trimeric Spike IgG levels (BAU/mL) at 9, 12 and 21 months post-primary vaccination among the groups of recruited vaccinated subjects. Sample size (*n*) and relative percentages are reported for each group at each time point.

Inter-group comparisons demonstrated that the heterologous ChAd/BNT/mRNA-1273 prime-boost regimen elicited the highest anti-Spike IgG levels at both 9 and 21 months post-primary vaccination compared to homologous immunization schedules (ChAd/ChAd/mRNA-1273 and ChAd/ChAd/BNT). Specifically, median titers for the ChAd/BNT/mRNA-1273 group were 3070 BAU/mL at month 9, declined to 1148 BAU/mL at month 12 and rebounded to 4647.5 BAU/mL by month 21. Similarly, the heterologous ChAd/BNT/BNT group maintained comparable IgG levels at months 9 and 12 (2020 BAU/mL and 2035 BAU/mL, respectively) but displayed a marked increase at month 21 (3915 BAU/mL) that surpassed titers observed in the homologous groups (~2400 BAU/mL). Notably, although not statistically significant (*p* = 0.0521), the ChAd/BNT/mRNA-1273 group demonstrated higher IgG levels compared to ChAd/ChAd/mRNA-1273 at the 21-month time point (**Table 2**, **Figure 1A**).

These results demonstrate the enhanced and more sustained humoral response elicited by heterologous vaccination schedules. particularly ChAd/BNT/mRNA-1273, compared to homologous regimens.

### 3.2 Impact of SARS-CoV-2 infection on humoral immunity

To evaluate the impact of viral infection on humoral immunity, anti-trimeric Spike IgG levels were compared between N+ and N− participants within each vaccination group. Notably, N+ individuals displayed significantly higher anti-Spike IgG titers compared to N− individuals across all time points, consistent with an infection-driven immune boost. For instance, within the ChAd/ChAd/mRNA-1273 group, IgG levels significantly decreased in N− individuals between 9 and 12 months (*p* < 0.0001) but increased between 12 and 21 months (*p* < 0.001) post-primary vaccination, likely due to the administration of a fourth vaccine dose in some participants or late natural infections (**Figure 1B**, **Table 3**, **Supplementary Table S5**). Similar trends were observed in the ChAd/BNT/BNT group, where N+ individuals consistently exhibited higher IgG titers than N− individuals, particularly at 21 months (*p* < 0.05).

**Table 3:** Distribution of subjects receiving BNT or mRNA-1273 as the fourth dose.

Vaccine Schedule	Subjects	4th dose				
		BNT		mRNA-1273		Total 4th dose
		N+	N-	N+	N-	
ChAd/ChAd/mRNA-1273	N+ = 34 N- = 22 Tot = 56	1/34 (2.9%) - 1/56 (1.8%)	- 3/22 (13.6%) 3/56 (5.4%)	3/34 (8.8%) - 3/56 (5.4%)	- 3/22 (13.6%) 3/56 (5.4%)	N+ = 4/34 (11.8%) N- = 6/22 (27.3%) Tot = 10/56’(17.9%)
ChAd/ChAd/BNT	N+ = 9 N- = 4 Tot = 13	4/9 (44.4%) - 4/13 (30.8%)	- 2/4 (50.0%) 2/13 (15.4%)	1/9 (11.1%) - 1/13 (7.7%)	- 0/4 (0.0%) 0/13 (0.0%)	N+ = 5/9 (55.6%) N- = 2/4 (50.0%) Tot = 7/13 (53.8%)
ChAd/BNT/mRNA-1273	N+ = 5 N- = 1 Tot = 6	0/5 (0.0%) - 0/6 (0.0%)	- 0/1 (0.0%) 0/6 (0.0%)	0/5 (0.0%) - 0/6 (0.0%)	- 1/1 (100.0%) 1/6 (16.7%)	N+ = 0/5 (0.0%) N- = 1/1 (100.0%) Tot = 1/6 (16.7%)
ChAd/BNT/BNT	N+ = 16 N- = 8 n = 24	1/16 (6.3%) - 1/24 (4.2%)	- 1/8 (12.5%) 1/24 (4.2%)	0/16 (0.0%) - 0/24 (0.0%)	- 0/8 (0.0%) 0/24 (0.0%)	N+ = 1/16 (6.3%) N- = 1/8 (12.5%) Tot = 2/24 (8.3%)

Distribution of subjects receiving BNT or mRNA-1273 as the fourth dose at 21 months post-primary vaccination, stratified by vaccination schedule and infection status (N+ = infected, N− = uninfected). Data are presented as the number of subjects over the total within each subgroup, with relative percentages provided in parentheses.

Intra-group analysis revealed a statistically significant difference (*p* < 0.0001) in anti-trimeric Spike IgG titers between N+ and N− individuals immunized with ChAd/ChAd/mRNA-1273 at the 12-month time point, with higher IgG titers observed in N+ subjects. Smaller but statistically significant differences (*p* < 0.05) were also detected at i) month 9 within the same group, ii) month 12 between N+ and N− individuals in the ChAd/BNT/BNT group, and iii) month 21 for the ChAd/BNT/mRNA-1273 group (**Figure 1B**). Non-significant trends in IgG differences between N+ and N− individuals were observed in the ChAd/BNT/BNT group at month 9 (*p* = 0.0731), ChAd/BNT/mRNA-1273 at month 12 (*p* = 0.1242), ChAd/ChAd/BNT at month 21 (*p* = 0.1063), and ChAd/BNT/BNT at month 21 (*p* = 0.1025).

When focusing on the magnitude of anti-trimeric Spike IgG responses, higher median antibody levels were consistently observed in N+ compared to N− individuals across both homologous and heterologous vaccination schedules (ChAd/ChAd/mRNA-1273, ChAd/BNT/mRNA-1273 and ChAd/BNT/BNT) at all study time points, with one exception. At 21 months, IgG levels in the ChAd/ChAd/mRNA-1273 group were comparable between N+ and N− individuals (**Supplementary Table S5**). In contrast, for the ChAd/ChAd/BNT group, anti-trimeric Spike IgG median levels remained comparable between N+ and N− subjects at 9 and 12 months, but an increased response was evident in N+ subjects at 21 months. These findings suggest that while infection significantly boosts antibody responses across all vaccination groups, the magnitude and duration of this boost may vary depending on the specific vaccination regimen and time since vaccination or infection.

### 3.3 Influence of fourth dose on humoral immunity

To assess the potential impact of a fourth vaccine dose on SARS-CoV-2 anti-trimeric Spike IgG titers, subjects were grouped based on their vaccination schedule and infection status. Although no statistical comparisons were performed due to the small number of participants receiving a fourth dose, descriptive analysis was provided. At 21 months, 20 individuals (20.2% of the cohort) received a fourth vaccine dose with variations depending on their vaccination schedule and infection status (**Supplementary Table S3**, **Table 3**). Among participants in the ChAd/ChAd/mRNA-1273 group, 2.9% of infected [N+] individuals received BNT, while 8.8% received mRNA-1273. Conversely, 27.3% of uninfected [N−] individuals in the same group received either BNT or mRNA-1273. Similarly, in the ChAd/ChAd/BNT group, fourth-dose administration was more frequent among uninfected subjects (50% for BNT), whereas 44.4% of infected individuals received BNT. In contrast, the ChAd/BNT/mRNA-1273 and ChAd/BNT/BNT groups showed limited fourth-dose uptake, with mRNA-1273 administered exclusively in uninfected individuals of the ChAd/BNT/mRNA-1273 group and BNT being the predominant choice in ChAd/BNT/BNT recipients.

### 3.4 Impact of homologous and heterologous priming on long-term anti-trimeric Spike IgG responses

To evaluate the impact of primary vaccination schedules (ChAd/ChAd vs ChAd/BNT) on long-term immune responses, we analyzed anti-trimeric Spike IgG levels at 9, 12, and 21 months post-primary vaccination. While this analysis focuses on the priming effect of homologous versus heterologous schedules, it is important to note that by month 21, participants had also received additional doses (third and, in some cases, fourth). These subsequent doses likely contributed to the observed recovery in IgG levels, as discussed in earlier sections. However, isolating the impact of the primary schedule provides valuable insights into the foundational immune response. Longitudinal analysis of the anti-trimeric Spike IgG response revealed a similar trend between homologous and heterologous primary vaccination schedules, characterized by a decline in antibody titers between months 9 and 12, followed by a recovery at month 21. In the homologous ChAd/ChAd schedule, IgG titers decreased significantly between months 9 and 12 (*p* < 0.001), from a median of 2050 BAU/mL (IQR: 39.3–41400) to 1495 BAU/mL (IQR: 39.2–17600), and then increased significantly at month 21 (*p* < 0.01) to a median of 2420 BAU/mL (IQR: 117–15260) (**Supplementary Figure S5A**, **Supplementary Table S6**). A similar, non-statistically significant decline was observed in the heterologous ChAd/BNT schedule, where IgG levels decreased from a median of 2280 BAU/mL (IQR: 605–40000) at month 9 to 1952 BAU/mL (IQR: 237–20600) at month 12 (*p* = 0.0679). By month 21, IgG levels in the ChAd/BNT group reached a median of 3920 BAU/mL (IQR: 165–14500), significantly higher than those observed in the ChAd/ChAd group (*p* < 0.01).

### 3.5 Longitudinal analysis of SARS-CoV-2 anti-Nucleocapsid at 9 12 and 21 months post-vaccination between homologous and heterologous schedule

Evaluation of IgM and IgG anti-Nucleocapsid (anti-N) antibodies in subjects vaccinated with ChAd/ChAd (homologous) and ChAd/BNT (heterologous) schedules revealed a comparable increase in the percentage of SARS-CoV-2 positive individuals over the study period. Specifically, positivity rates increased from 22.4% to 62.3% in the ChAd/ChAd group and from 26.3% to 70.0% in the ChAd/BNT group between months 9, 12, and 21 (**Supplementary Figure S6**).

Longitudinal analysis of anti-trimeric Spike IgG titers among infected [N+] and uninfected [N−] individuals within the ChAd/ChAd and ChAd/BNT groups highlighted statistically significant differences, particularly in N− participants (**Supplementary Figure S5B**). In the ChAd/ChAd group, anti-trimeric Spike IgG titers in N− individuals showed a significant decrease between months 9 and 12 (*p* < 0.0001), followed by a significant increase at month 21 (*p* < 0.01). In contrast. N− individuals in the ChAd/BNT group exhibited a smaller, non-significant decrease in IgG titers between months 9 and 12 (*p* = 0.0535).

Intra-group comparisons revealed significant differences in IgG titers between N+ and N− individuals at months 9 and 12 for both vaccination schedules. At month 12, the differences were more pronounced in the ChAd/ChAd group (*p* < 0.0001) compared to the ChAd/BNT group (*p* < 0.01). At month 9, smaller but significant differences (*p* < 0.05) were observed between N+ and N− individuals for both schedules. By month 21, N+ participants in the ChAd/BNT group had significantly higher IgG titers compared to N+ individuals in the ChAd/ChAd group (*p* < 0.01).

Focusing on IgG levels, the highest median titers across all time points were consistently observed in N+ individuals vaccinated with ChAd/BNT, with values of 6195 BAU/mL at month 9, 4220 BAU/mL at month 12, and 3925 BAU/mL at month 21 (**Supplementary Figure S5B**, **Table 4**). In contrast, N− individuals in both vaccination groups had the lowest median antibody titers, ranging from 1144 BAU/mL (ChAd/ChAd at month 12) to 3480 BAU/mL (ChAd/BNT at month 21). Notably, at month 21, N− participants in the ChAd/BNT group showed a median IgG titer (3480 BAU/mL) comparable to that of N+ participants (3925 BAU/mL), suggesting a robust recovery of antibody levels in this subgroup.

**Table 4:** SARS-CoV-2 anti-trimeric Spike IgG titers by primary vaccination schedule, time points and infection status.

		ChAd/ChAd		ChAd/BNT	
		N+	N-	N+	N-
IgG titer (BAU/mL)	9 months Median (IQR)	n = 30 (15.4%) 3420 (579-25600)	n = 104 (53.3%) 2040 (39.3-41400)	n = 16 (8.2%) 6195 (725-40000)	n = 45 (23.1%) 1930 (605-22200)
	12 months Median (IQR)	n = 48 (27.9%) 2500 (123-17600)	n = 75 (43.6%) 1144 (39.2-10760)	n = 23 (13.4%) 4220 (237-10340)	n = 26 (15.1%) 1275 (287-20600)
	21 months Median (IQR)	n = 43 (43.4%) 2650 (333-12240)	n = 26 (26.3%) 2345 (117-15260)	n = 21 (21.2%) 3925 (1440-14500)	n = 9 (9.1%) 3480 (165-6050)

Median and interquartile range (IQR) of SARS-CoV-2 anti-trimeric Spike IgG levels in ChAd/ChAd and ChAd/BNT immunized subjects at 9, 12, and 21 months post-vaccination. stratified by infection status (N+ = infected. N− = uninfected). Data are presented as median IgG titers (BAU/mL) with IQR (25^th^-75^th^) in parentheses, alongside the number and percentage of subjects within each group.

### 3.6 Spike-specific memory B-cells induction at 21 months post-vaccination

**Figure 2:**
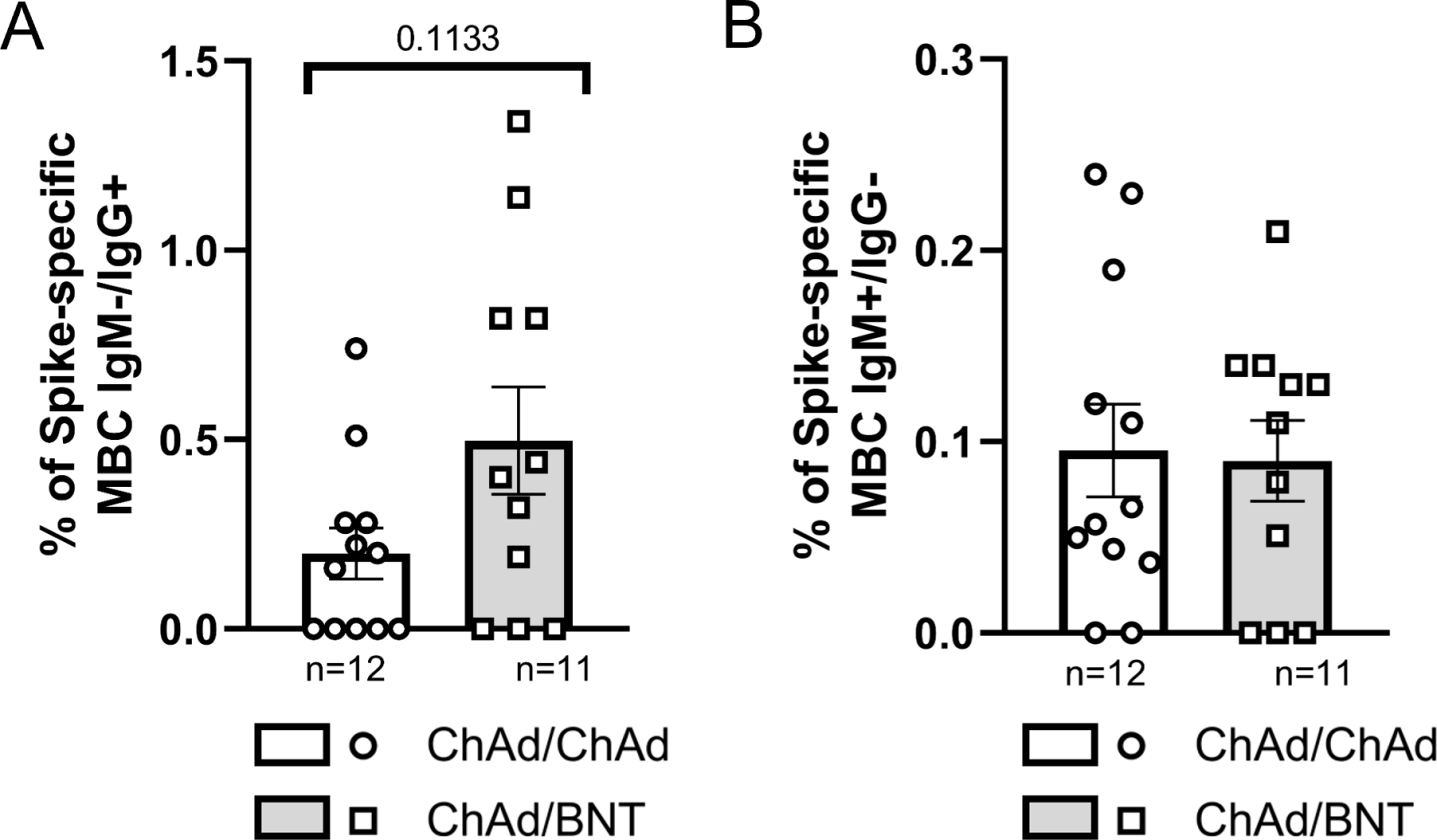
Inter-group comparison of the percentage of Spike-specific memory B-cells [MBCs] among four vaccinated groups at 21 months post-vaccination. **(A)** Percentages of IgM−/IgG+ MBCs. **(B)** Percentages of IgM+/IgG− MBCs. Bars represent the mean with standard error of the mean [SEM]. The number of subjects analyzed in each group is reported below the x-axis. Statistical significance was assessed using the Kruskal-Wallis test with Dunn’s *post hoc* comparison and Mann-Whitney U-test (*p* < 0.05).

To investigate the cellular response induced by different vaccination strategies, the percentages of Spike-specific memory B-cells [MBCs] were analyzed in PBMCs collected 21 months post-primary vaccination. Although not statistically significant, participants who received heterologous vaccination schedules (ChAd/BNT/mRNA-1273 and ChAd/BNT/BNT) displayed higher percentages of IgM−/IgG+ MBCs compared to those vaccinated with homologous schedules (ChAd/ChAd/mRNA-1273 and ChAd/ChAd/BNT) (**Figure 2A**). Notably, the largest difference was observed between ChAd/ChAd/mRNA-1273 and ChAd/BNT/BNT (*p* = 0.1699). For IgM+/IgG− MBCs, a significant increase (*p* < 0.05) was detected in ChAd/ChAd/BNT subjects compared to ChAd/ChAd/mRNA-1273 participants (**Figure 2B**). A similar but non-significant trend was observed when comparing ChAd/ChAd/mRNA-1273 to ChAd/BNT/BNT (*p* = 0.1395) (**Figure 2B**). Overall, IgM−/IgG+ MBCs were more prevalent than IgM+/IgG− MBCs across all vaccination strategies. Despite the variability among groups, heterologous vaccination schedules demonstrated a tendency to elicit stronger memory B-cell responses compared to homologous immunization.

### 3.7 Spike-specific memory B-cells induction at 21 months following homologous or heterologous primary vaccination

**Figure 3:**
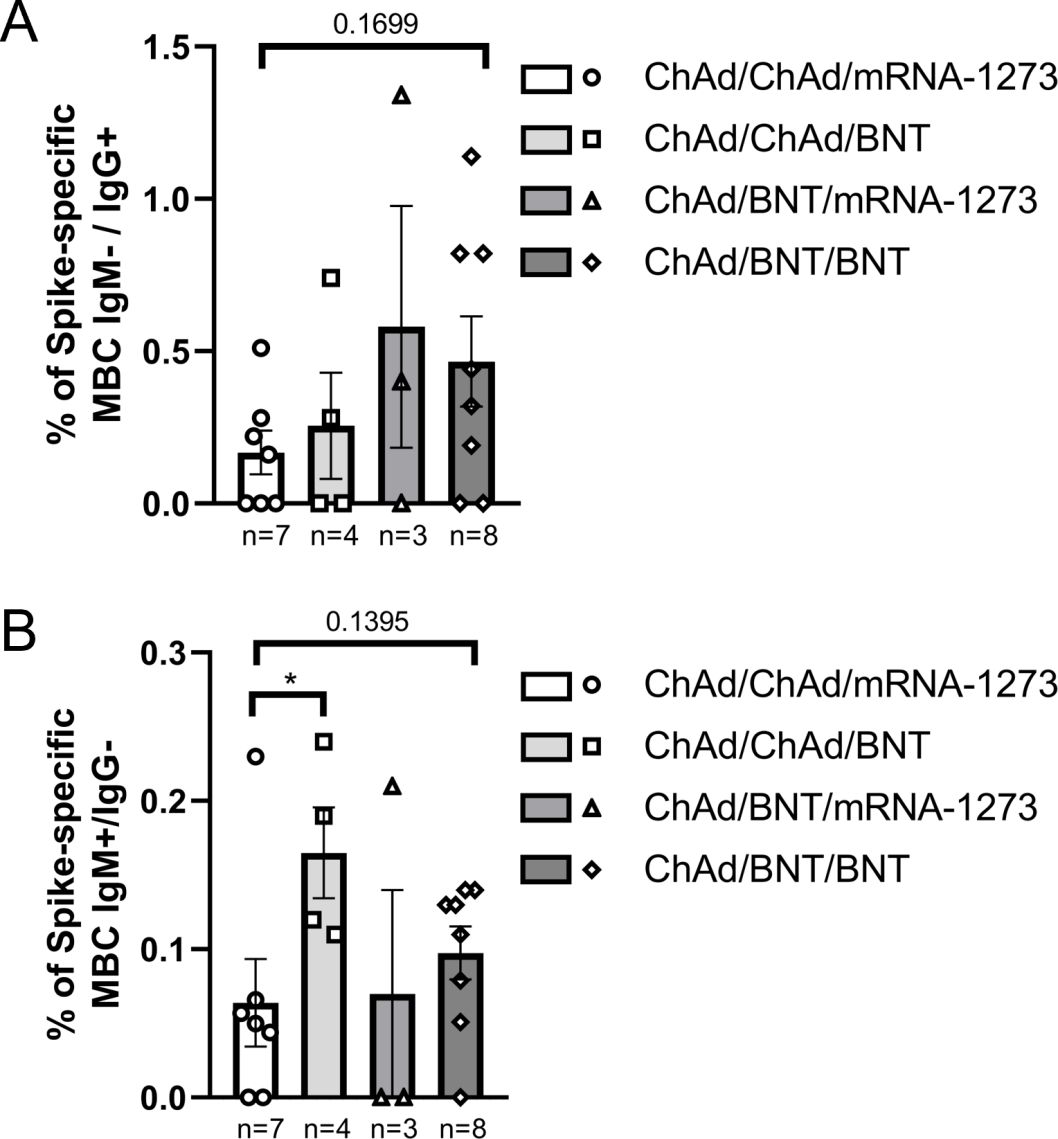
Inter-group comparison of the percentage of Spike-specific memory B-cells [MBCs] between subjects receiving homologous (ChAd/ChAd) and heterologous (ChAd/BNT) primary vaccination schedules at 21 months post-vaccination. **(A)** Percentages of IgM−/IgG+ MBCs. **(B)** Percentages of IgM+/IgG− MBCs. Bars represent the mean with standard error of the mean [SEM]. The number of subjects analyzed in each group is reported below the x-axis. Statistical significance was assessed using the Mann-Whitney U-test. *p < 0.05.

Comparison of Spike-specific IgM−/IgG+ memory B-cells [MBCs] responses between subjects receiving homologous (ChAd/ChAd) and heterologous (ChAd/BNT) primary vaccination schedules revealed a higher, though not statistically significant response in the heterologous group (*p* = 0.1133) (**Figure 3A**). In contrast, comparable levels of IgM+/IgG− MBCs were observed between the two groups (**Figure 3B**). Notably, the percentages of IgM−/IgG+ MBCs were consistently higher than those of IgM+/IgG− MBCs across both vaccination strategies. The mean values were 0.2% (ChAd/ChAd) and 0.5% (ChAd/BNT) for IgM−/IgG+ MBCs, compared to 0.1% (ChAd/ChAd) and 0.09% (ChAd/BNT) for IgM+/IgG− MBCs.

Further intra-group analysis at 21 months stratified by infection status (N+ = infected, N− = uninfected) showed a trend toward higher IgM−/IgG+ MBCs induction in infected [N+] subjects compared to uninfected [N−] within the ChAd/BNT group, though this difference did not reach statistical significance (*p* = 0.0563) (**Supplementary Figure S7A**). Moreover, within infected participants, the ChAd/BNT group exhibited an increased IgM−/IgG+ MBC response compared to the ChAd/ChAd group (*p* = 0.1212). Conversely, levels of IgM+/IgG− MBCs were similar across infection statuses and vaccination strategies (**Supplementary Figure S7B**).

### 3.8 Spike-specific T-cells induction at 21 months post-vaccination

**Figure 4:**
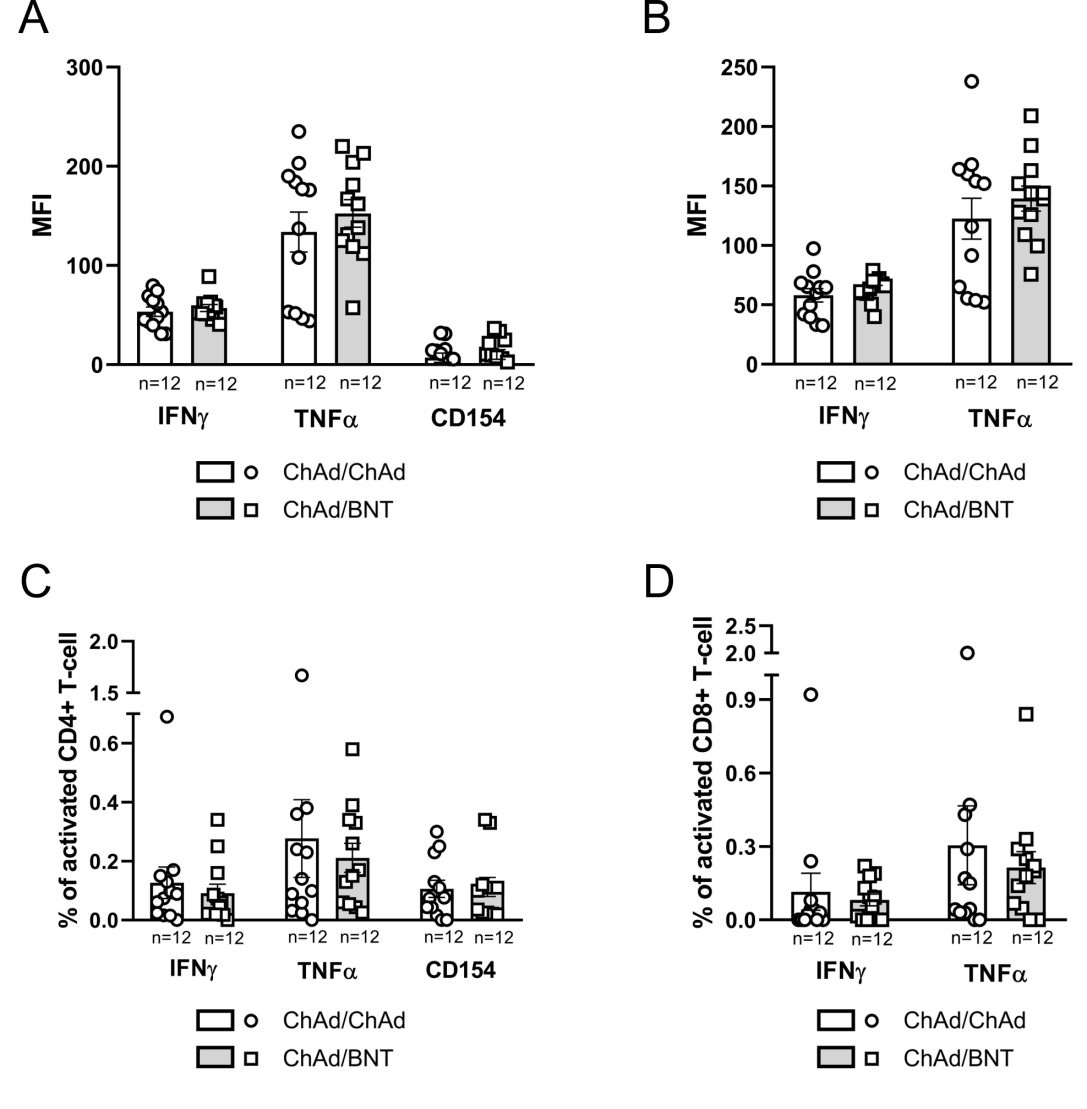
Inter-group comparison of the intracellular cytokine expression in CD4+ and CD8+ T-cells across four vaccination groups at 21 months post-vaccination. **(A)** Mean fluorescence intensity [MFI] of IFNγ, TNFα, and CD154 expression in CD4+ T-cells. **(B)** MFI of IFNγ and TNFα expression in CD8+ T-cells. **(C)** Percentage of CD4+ T cells expressing IFNγ, TNFα, or CD154. **(D)** Percentage of CD8+ T cells expressing IFNγ or TNFα. Bars represent the mean with standard error of the mean [SEM]. Sample sizes for each group are reported below the x-axis. Statistical significance was assessed using the Kruskal-Wallis test with Dunn’s *post hoc* multiple comparisons.

**Figure 5:**
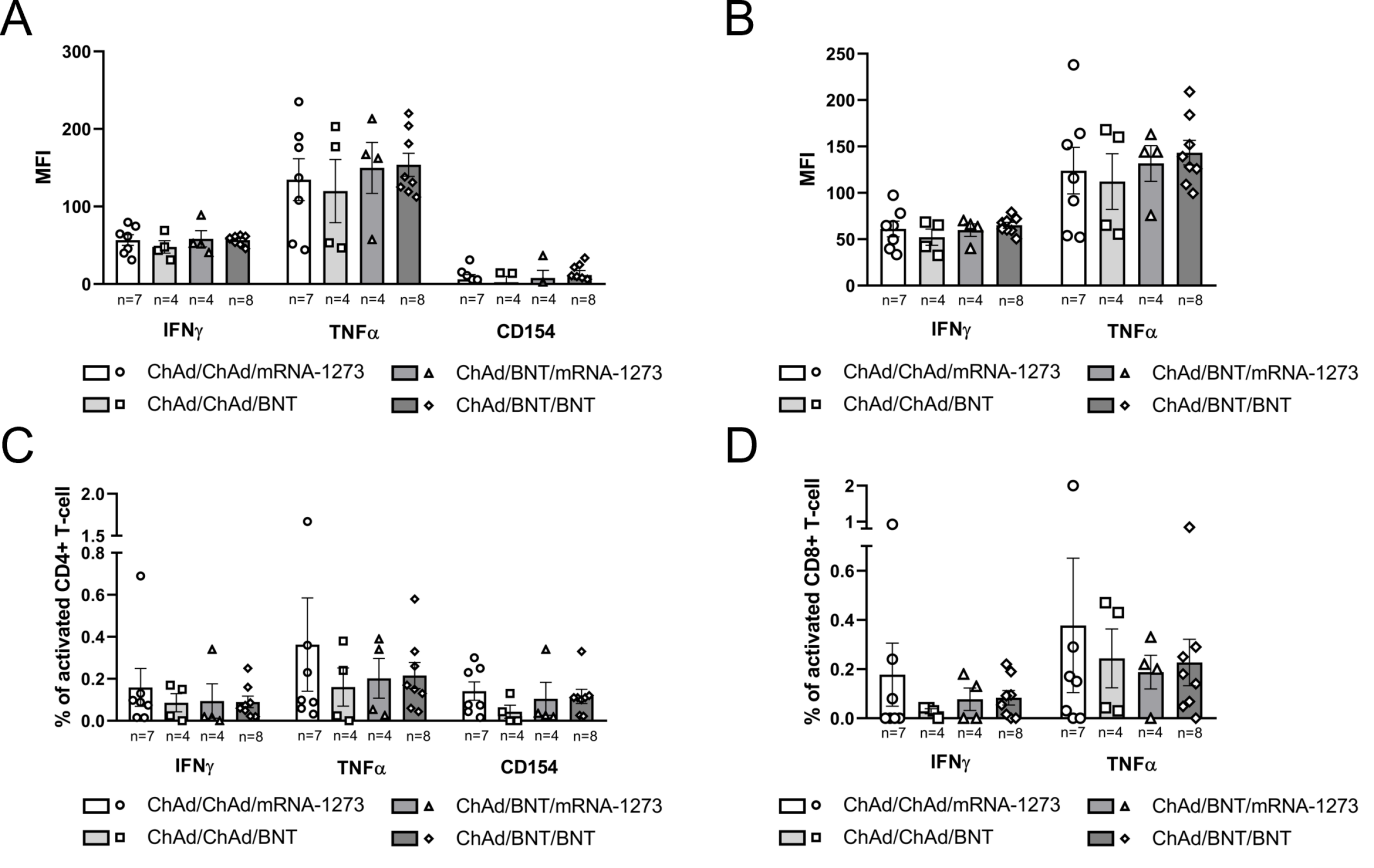
Inter-group comparison of the intracellular cytokine expression in CD4+ and CD8+ T-cells between homologous (ChAd/ChAd) and heterologous (ChAd/BNT) primary vaccination schedules at 21 months post-vaccination. **(A)** Mean fluorescence intensity [MFI] of IFNγ, TNFα and CD154 expression in CD4+ T-cells. **(B)** MFI of IFNγ and TNFα expression in CD8+ T-cells. **(C)** Percentage of CD4+ T cells expressing IFNγ, TNFα, or CD154. **(D)** Percentage of CD8+ T cells expressing IFNγ or TNFα. Bars represent the mean with standard error of the mean [SEM]. Sample sizes for each group are reported below the x-axis. Statistical significance was assessed using the Mann-Whitney U test.

Intracellular cytokine expression by T-cells isolated from PBMCs of vaccinated subjects was evaluated at 21 months post-immunization. Comparable levels of IFNγ, TNFα, and CD154 expression by CD4+ T-cells, as well as IFNγ and TNFα expression by CD8+ T-cells, were observed across the four vaccination groups: ChAd/ChAd/mRNA-1273, ChAd/ChAd/BNT, ChAd/BNT/mRNA-1273 and ChAd/BNT/BNT (**Figures 4A, B**). When subjects were grouped according to their homologous or heterologous primary vaccination schedules, no statistically significant differences were detected in the expression of the same T-cell cytokines (i.e. IFNγ, TNFα and CD154 for CD4+ T-cells and IFNγ and TNFα for CD8+ T-cells) (**Figures 5A, B**). Intra-group analysis based on infection status (N+ = infected; N− = uninfected) within the ChAd/ChAd and ChAd/BNT groups showed no significant differences in CD4+ T-cell cytokine expression (IFNγ, TNFα and CD154) between infected [N+] and uninfected [N−] individuals (**Supplementary Figure S8A**). However, a trend toward higher mean cytokine expression, particularly IFNγ and TNFα, was observed in infected individuals compared to uninfected participants within the same vaccination group. For CD8+ T-cells, a statistically significant difference in TNFα expression was identified between infected [N+] and uninfected [N−] subjects within the ChAd/BNT group (*p* < 0.05) (**Supplementary Figure S8B**). Additionally, although not statistically significant, higher TNFα expression was observed in N+ heterologous primary vaccinated subjects compared to N+ homologous immunized individuals (*p* = 0.1717). Similarly, increased IFNγ expression in CD8+ T-cells was noted in N+ ChAd/BNT subjects compared to other groups, but this difference did not reach statistical significance (*p* = 0.1490), (**Supplementary Figure S9**). While the overall trend of activation across vaccination groups were similar, MFI-based analysis highlighted differences in cytokine expression intensity that were not evident when analyzing only the percentage of cytokine-positive cells. This discrepancy likely reflects the increased expression level per cell, rather than an increased frequency of positive cells, and suggests that MFI better captures the magnitude of T-cell activation, particularly in the context of previous infection (**Figures 4C, D**, **5C, D**, **Supplementary Figure S8C, D**). Polyfunctionality analysis of cytokine-expressing T cells revealed no statistically significant differences among the four vaccine groups (**Supplementary Figure S9**), between homologous (ChAd/ChAd) and heterologous (ChAd/BNT) primary regimens (**Supplementary Figure S10**), or between infected and uninfected individuals within these groups (**Supplementary Figure S11**). Overall, these results indicate that both homologous and heterologous vaccination regimens induced persistent T-cell responses, with a notable enhancement of TNFα production in CD8+ T-cells among SARS-CoV-2 infected individuals who received a heterologous primary vaccination schedule

## 4 Discussion

The COVID-19 pandemic has necessitated rapid vaccine development and the implementation of diverse immunization strategies, including homologous and heterologous schedules, as well as additional booster doses (5, 12–15). Notwithstanding the interest on the development of increasingly new SARS-CoV-2 vaccines able to adapt and respond to the emerging variants of concerns, there remains limited research on the long-term durability of immune responses induced by different vaccination strategies (16–18).

Our previous studies focused on the evaluation of heterologous and homologous COVID-19 vaccination up to six months after primary immunization (8, 9). Analysis of the humoral response highlighted a stronger anti-viral immune induction for the heterologous ChAd/BNT vaccination compared to the homologous ChAd/ChAd at 2-, 4- and 6-months after the primary immunization. Additional studies have further highlighted the efficacy of heterologous vaccination schedules. For example, highest titers of SARS-CoV-2 anti-S IgG levels and increased T-cell responses were reported in BNT/ChAd and ChAd/BNT vaccinated healthy adults compared to the homologous ChAd/ChAd group (3). Similarly, higher and longer-lasting anti-RBD antibody levels were observed in heterologous ChAd/Coronavac vaccination schedules when compared to homologous ChAd/ChAd regimens (19).

In this study, we extended the observation period to 21 months post-primary vaccination, providing a long-term analysis of both humoral and cellular immune responses following SARS-CoV-2 vaccination. We compared homologous (ChAd/ChAd) and heterologous (ChAd/BNT) vaccination schedules in participants who also received a third mRNA booster dose (BNT or mRNA-1273). Moreover, due to the extended duration of this study, participants were exposed to SARS-CoV-2 variants of concern circulating during the study period, likely including Delta, which was dominant at the start of the study, and Omicron which emerged and became the prevailing variant by late 2021 and early 2022. Also, some participants were already SARS-CoV-2 positive at the first time point (9 months after primary vaccination), suggesting earlier encounters with the virus. This prior exposure may have included other variants, such as Beta or Gamma, potentially influencing the baseline immune responses and contributing to the observed variability over time. It is also important to note that the vaccines used in this study—Pfizer-BioNTech (BNT162b2), AstraZeneca (ChAdOx1-S), and Moderna (mRNA-1273)—were all based on the spike protein of the original Wuhan-Hu-1 strain, which may further influence immune recognition and responses to emerging variants (20). The longitudinal analysis of anti-S IgG titers revealed a general trend across all groups: antibody levels peaked at 9 months (three months post-booster), declined by 12 months and increased again by 21 months. The elevated antiviral antibody concentration observed at 9 months can be attributed to immune system activation following the third dose of SARS-CoV-2 vaccine, as samples were collected approximately three months post-booster (21, 22). Booster vaccination is known to robustly enhance antibody levels, peaking within the first 1–3 months after administration, before gradually declining over time due to the natural waning of plasma cell activity (23, 24). Finally, the increase in anti-S IgG observed at 21 months is likely a result of natural SARS-CoV-2 infections acting as immune boosters, alongside the administration of a fourth vaccine dose at approximately month 16, which further stimulated humoral immune responses (25, 26).

Our findings demonstrate that heterologous vaccination with ChAd/BNT, followed by an mRNA booster, elicits a more robust and sustained humoral response compared to homologous ChAd/ChAd vaccination. This is evident in the higher anti-S IgG titers observed in the heterologous groups, particularly at the 21-month time point. These results align with previous studies that have shown the benefits of heterologous prime-boost strategies for COVID-19 and other infectious diseases (3, 27, 28). In contrast, the homologous ChAd/ChAd/mRNA-1273 group was the only group to show a significant increase in anti-S IgG levels between months 9 and 12, followed by a significant decrease from months 12 to 21. These findings highlight a differential impact of homologous and heterologous primary vaccination schedules on the durability and dynamics of the immune response, consistent with previous observations (5).

Given the extended study period, we analyzed additional variables that could influence anti-viral antibody production, such as SARS-CoV-2 infection and the administration of a fourth vaccine dose. When evaluating the percentage of SARS-CoV-2 infected [N+] and uninfected [N−] individuals, we observed a general increase in the number of infected participants from month 9 to month 21, independently of the vaccination schedule., This trend indicates that infection rates increased across all groups over time, with the ChAd/BNT/mRNA-1273 group showing the highest percentages of infected individuals at both 12 and 21 months. While this observation may suggest differences in exposure or other external factors, all vaccination schedules contributed to a sustained level of immune protection over the study period.

When comparing anti-S IgG titers between N+ and N− subjects within each vaccination group, notable differences emerged. At all-time points, N+ individuals exhibited higher anti-S IgG levels compared to N− individuals, likely due to a ‘booster effect’ induced by SARS-CoV-2 infection (29). In addition, we observed significant differences within specific vaccination groups at later time points. For instance, in the ChAd/ChAd/mRNA-1273 group, a significant increase in anti-S IgG titers was seen in N− individuals between months 12 and 21, likely driven by the fourth vaccine dose administered at month 16 (approximately 30% of N− individuals in this group received BNT or mRNA-1273 as a fourth dose). Similarly. in the ChAd/ChAd/BNT group, the higher anti-S IgG titers observed in N+ individuals compared to N− participants at month 21 may reflect a ‘double booster effect’ resulting from both SARS-CoV-2 infection and the fourth vaccine dose. Notably, around 50% of participants in this group—regardless of infection status—received a fourth dose (BNT or mRNA-1273) at month 16.

A significant variability in anti-S IgG titers was observed over the study period, particularly in the ChAd/ChAd/mRNA-1273 group. Specifically: i) a significant decrease in antibody levels occurred between months 9 and 12, followed by an increase between months 12 and 21; ii) at month 21, anti-S IgG titers in this group were lower compared to those in the ChAd/BNT/BNT group; and iii) significant differences between N+ and N− individuals were noted at months 9 and 12, but these differences were no longer observed at month 21. Conversely, in the ChAd/BNT/BNT group, higher anti-S IgG titers were observed for N+ subjects compared to N− subjects, but only at the 12-month time point.

To further clarify the impact of primary vaccination on the immune response, we grouped participants based on homologous (ChAd/ChAd) or heterologous (ChAd/BNT) regimens, increasing the statistical power of the analysis. Significant fluctuations were observed in the homologous schedule, with a marked decrease in IgG levels from 9 to 12 months followed by a rebound at 21 months, a pattern particularly evident in N− individuals and likely influenced by the ChAd/ChAd/mRNA-1273 group. In contrast, the heterologous schedule showed more stable IgG levels over time, with consistently higher antibody titers compared to the homologous group, especially at the later time points.

Despite these variations, both the ChAd/ChAd and ChAd/BNT schedules demonstrated comparable trends in infection rates over time, as indicated by similar increases in the percentage of N+ subjects. While these findings suggest that the overall immunization capacity of both schedules is similar in preventing SARS-CoV-2 infection, homologous vaccination may result in a lower production of long-lived plasma cells compared to heterologous vaccination, potentially explaining the observed differences in antibody persistence (25, 30).

Finally, we further investigated the cellular immune response to the different vaccination schedules in a subgroup of participants equally distributed between N+ and N− individuals who had received either homologous or heterologous primary vaccination. At 21-month post-primary immunization, analysis of both B- and T-cell populations revealed the persistence of anti-S specific memory B cells [MBCs] and T-cells. This persistence extends beyond the 6–8 months typically reported following COVID-19 vaccination or natural infection (31–33). Higher percentages of anti-S specific IgM⁻/IgG⁺ MBCs were observed in participants who received the heterologous primary ChAd/BNT vaccination schedule compared to those who received the homologous ChAd/ChAd schedule, in both two-dose and three-dose groups. Additionally, an increased proportion of anti-viral IgM⁻/IgG⁺ MBCs was detected in SARS-CoV-2 infected [N+] individuals within both ChAd/ChAd and ChAd/BNT groups, underscoring the ‘booster effect’ of natural viral infection in reactivating the immune system (26). In contrast, low and variable percentages of IgM⁺/IgG⁻ MBCs were observed across the vaccinated groups. This is likely due to their early emergence during the initial months following immunization, followed by a gradual class-switching to IgM⁻/IgG⁺ MBCs over the course of the study period (31). Regarding CD4⁺ and CD8⁺ T-cell responses, comparable levels of anti-S specific cytokine production were observed across the vaccinated groups, regardless of the number of doses administered. However, higher mean expression of IFN-γ and TNF-α by CD8⁺ T-cells was particularly evident in SARS-CoV-2 infected [N+] individuals within the ChAd/BNT group compared to uninfected [N-] subjects. This finding suggests a preferential cytotoxic activation of the immune system as a consequence of natural SARS-CoV-2 infection (34) While this study provides valuable insights into the long-term immune responses following different COVID-19 vaccination strategies, certain limitations should be acknowledged. The sample size, while sufficient for the primary analyses, could be expanded in future studies to enhance the statistical power, especially when analyzing subgroups based on infection status (N+ and N-) or the administration of a fourth vaccine dose. Additionally, the reliance on anti-nucleocapsid antibody testing at specific time points to determine infection status may have resulted in some misclassification of individuals with prior asymptomatic infections. Incorporating more frequent testing and exploring other markers of infection could improve the accuracy of infection status determination in future investigations. Furthermore, the predominantly young to middle-aged adult cohort from a single region in Italy may not fully represent the broader population. Therefore, caution should be exercised when generalizing these findings to other age groups or geographical locations. Despite these limitations, the study’s longitudinal design, detailed immunological assessments, and inclusion of both homologous and heterologous vaccination schedules provide valuable data on the persistence and nature of immune responses following COVID-19 vaccination and infection.

## 5 Conclusions

This study provides a comprehensive, real-world assessment of long-term immune responses following homologous and heterologous COVID-19 vaccination, analyzing data from an Italian cohort up to 21 months post-primary immunization. Our results demonstrate that both homologous (ChAd/ChAd) and heterologous (ChAd/BNT) vaccination strategies, particularly when followed by an mRNA booster, induce robust and persistent humoral and cellular immunity, including anti-Spike IgG, memory B cells, and T cells. Notably, the heterologous ChAd/BNT regimen, boosted with an mRNA vaccine, elicited a significantly stronger and more durable immune response compared to the homologous ChAd/ChAd schedule, particularly in terms of long-term antibody persistence and cellular immune activation. Furthermore, we found that natural SARS-CoV-2 infection significantly enhanced both humoral and cellular immune responses, acting as a natural booster and potentially broadening protection. These findings underscore the effectiveness of heterologous vaccination in achieving robust long-term immunity and highlight the significant impact of hybrid immunity. Future studies should focus on larger, more diverse cohorts to further validate these findings and investigate the durability of responses beyond 21 months. Moreover, further research is needed to elucidate the optimal timing and frequency of booster doses, particularly in the context of emerging variants and varying infection histories. Such studies will be crucial to inform future vaccine development and refine immunization policies for achieving long-term population protection against COVID-19.

## Data Availability

The original contributions presented in the study are included in the article/**Supplementary Material**. Further inquiries can be directed to the corresponding author/s.
