# Supplementary material for: Analysis of humoral and cellular immune activation up to 21 months after heterologous and homologous COVID-19 vaccination
Source: Front Immunol. 2025 May 2;16:1579163. doi: 10.3389/fimmu.2025.1579163 (PMC12081374; doi:10.3389/fimmu.2025.1579163)
Supplement: Supplementary file 1 [file DataSheet1.docx]

Supplementary Material

**Table S1.** Timelines and booster schedules for recruited vaccinated subjects.

**Table S2.** Relationship among clinical and demographic parameters and vaccination schedule.

**Table S3.** Demographic characteristics of participants receiving a fourth COVID-19 vaccine dose.

**Table S4.** Relationship between infected [N+] and uninfected [N-] subjects along the follow-up period of study.

**Table S5.** SARS-CoV-2 anti-trimeric Spike IgG titers by vaccination schedule, time points and infection status.

**Figure S1:** Percentages of subjects vaccinated with mRNA-1273 or BNT as third dose at month 9, 12 and 21.

**Figure S2.** Flow diagram illustrating participant recruitment and specimen collection at each time-point and across multiple time points throughout the study.

**Figure S3.** Percentages of SARS-CoV-2 infected/uninfected (i.e. N+ or N-, respectively) subjects vaccinated with four different administration schedules at 9, 12 and 21 months after vaccination.

**Figure S4.** Representative plots of the gating strategy used to identify SARS-CoV-2 specific T cell and B cell populations. A) Gating strategy for quantifying intracellular cytokine expression in CD4+ or CD8+ T-cells; Lymphocyte gate > DEAD/CD14-/CD20- > CD3+ > CD4+ or CD8+ > mean fluorescence intensity [MFI] and percentage of TNF-α+, IFN-γ+, or CD154+ cells. Plots are shown as pseudocolor, arrows indicate sequential steps corresponding to the data in figure 4 and 5. B) Gating strategy to calculate the percentage of Spike specific MBC: Lymphocyte gate > Single cells > Live/Dead- > CD19+ > CD27+ > IgG+/IgM- or IgG-/IgM+ > Spike-Protein-PE+/PE-Vio® 770+. Plots are shown as pseudocolor, arrows indicate sequential steps corresponding to the data in figure 3.

**Figure S5****.** Inter-group comparison of SARS-CoV-2 anti-trimeric Spike protein IgG among ChAd/ChAd and ChAd/BNT vaccinated subjects at 9. 12 and 21 months post-primary vaccination. A) IgG levels in all subjects across the two vaccination schedules. B) IgG levels stratified by infection status (N+ = infected, N− = non-infected). Boxplots represent the median and interquartile range [IQR], with whiskers indicating the lowest and highest values according to Tukey-style plotting. The dotted red line denotes the upper limit of IgG anti-S quantification (2080 BAU/mL). Sample sizes for each group are reported below the x-axis. Kruskal Wallis test with Dunn’s post hoc multiple comparison and t test with Mann-Whitney post hoc pair-wise comparison. *p<0.05. **p < 0.01; ***P < 0.001; **** p < 0.0001.

**Figure S6.** Percentages of SARS-CoV-2 infected/uninfected (i.e. N+ or N-, respectively) subjects vaccinated with ChAd/ChAd or ChAd/BNT schedule at 9, 12, 21 months after vaccination.

**Figure S7****.** Inter-group comparison of the percentages of Spike specific memory B-cells [MBC] in SARS-CoV-2 infected/uninfected (i.e. N+ or N-, respectively) subjects vaccinated with ChAd/ChAd or ChAd/BNT schedule at 21 months after vaccination. A) Percentages of IgM- and IgG+ MBC. B) percentages of IgM+ and IgG- MBC. Bars represent the mean with standard error of the mean [SEM]. The number of subjects analyzed in each group is reported below the x-axis. Statistical significance was assessed using the Mann-Whitney U test.

**Figure S8.** Inter-group comparison of intracellular cytokine expression in CD4+ and CD8+ T-cells in SARS-CoV-2 infected/uninfected (i.e. N+ or N-, respectively) subjects vaccinated with ChAd/ChAd or ChAd/BNT schedule at 21 months after vaccination. A) Mean fluorescence intensity [MFI] of IFNγ, TNFα, and CD154 expression in CD4+ T-cells. B) MFI of IFNγ and TNFα expression in CD8+ T-cells. C) Percentage of CD4+ T-cells expressing IFNγ, TNFα, or CD154. D) Percentage of CD8+ T-cells expressing IFNγ or TNFα. Bars represent the mean with standard error of the mean (SEM). The number of subjects analyzed in each group is reported below the x-axis. Statistical significance was assessed using the Mann-Whitney U test; *p < 0.05.

**Figure S9.** Inter-group comparison of the percentage of polyfunctional intracellular cytokine-expressing CD4+ or CD8+ T-cells among four vaccinated groups at 21 months post-vaccination. A) Percentage of CD4+ T-cells co-expressing IFNγ/CD154, TNFα/CD154 or IFNγ/TNFα. B) Percentage of CD8+ T-cells co-expressing IFNγ/TNFα. Bars represent the mean with standard error of the mean [SEM]. Sample sizes for each group are reported below the x-axis. Statistical significance was assessed using the Kruskal-Wallis test with Dunn’s post hoc multiple comparisons.

**Figure S10**. Inter-group comparison of the percentage of polyfunctional intracellular cytokine-expressing CD4+ or CD8+ T-cells between subjects receiving homologous (ChAd/ChAd) and heterologous (ChAd/BNT) primary vaccination schedules at 21 months post-vaccination. A) Percentage of CD4+ T-cells co-expressing IFNγ/CD154, TNFα/CD154 or IFNγ/TNFα. B) Percentage of CD8+ T-cells co-expressing IFNγ/TNFα. Bars represent the mean with standard error of the mean [SEM]. Sample sizes for each group are reported below the x-axis. Statistical significance was assessed using the Mann-Whitney U test.

**Figure S11.** Inter-group comparison of the percentage of polyfunctional intracellular cytokine-expressing CD4+ or CD8+ T-cells in SARS-CoV-2 infected/uninfected (i.e. N+ or N-, respectively) subjects vaccinated with the ChAd/ChAd or ChAd/BNT schedule at 21 months after vaccination. A) Percentage of CD4+ T-cells co-expressing IFNγ/CD154, TNFα/CD154 or IFNγ/TNFα. B) Percentage of CD8+ T-cells co-expressing IFNγ/TNFα. Bars represent the mean with standard error of the mean (SEM). The number of subjects analyzed in each group is reported below the x-axis. Statistical significance was assessed using the Mann-Whitney U test; *p < 0.05.

**Table S1.** Timelines and booster schedules for recruited vaccinated subjects.

| **Vaccine schedule** | | | **Follow-up after primary vaccination** | | |
| --- | --- | --- | --- | --- | --- |
| **Primary** | | **Booster**  (days, mean ± SD) | **Months 9**  (days, mean ± SD) | **Months 12**  (days, mean ± SD) | **Months 21**  (days, mean ± SD) |
| **All** |  | | 274 ± 8 | 364 ± 8 | 631 ± 13 |
| **ChAd/ChAd** | mRNA-1273 (192 ± 13)  BNT (199 ± 15) | | 275 ± 7 | 364 ± 7 | 638 ± 5 |
|  |  |  | 277 ± 13 | 368 ± 14 | 638 ± 5 |
| **ChAd/BNT** | | mRNA-1273 (180 ± 14)  BNT (183 ± 16) | 273 ± 4  271 ± 9 | 365 ± 4  362 ± 8 | 617 ± 3  614 ± 10 |
| ChAd/ChAd refers to the ChAdOx1 COVID-19 vaccine [ChAd] administered as both the first and second doses (primary immunization), while ChAd/BNT refers to ChAd as the first dose and the BNT162b2 COVID-19 vaccine [BNT] as the second dose (primary immunization). Within brackets, days described as mean ± standard deviation [SD], represent days after primary immunization. | | | | | |

**Table S2.** Relationship among clinical and demographic parameters and vaccination schedule.

| A. Two doses vaccination groups | | | |
| --- | --- | --- | --- |
| Variable | **Vaccine Schedule** | | **p-value** |
|  | **ChAd/ChAd** | **ChAd/BNT** |  |
| Gender Male/Female | 63/76 | 29/36 | 0.9245 |
| Age median (IQR) | 55.0 (30.0-72.0) | 53.0 (26.0-62.0) | * |
| BMI median (IQR) | 24.1 (16.2-37.6) | 24.2 (16.7-36.7) | 0.9391 |

| B. Three doses vaccination groups | | | | | |
| --- | --- | --- | --- | --- | --- |
| Variable | **Vaccine Schedule** | | | | **p-value** |
|  | **ChAd/ChAd/**  **mRNA-1273** | **ChAd/ChAd/**  **BNT** | **ChAd/BNT/**  **mRNA-1273** | **ChAd/BNT/**  **BNT** |  |
| Gender Male/Female | 54/62 | 9/14 | 8/9 | 21/27 | 0.9208 |
| Age median (IQR) | 55.5  (30.0-71.0) | 55.0  (32.0-72.0) | 54.0  (26.0-61.0) | 52.0  (32.0-62.0) | >0.9999 |
| BMI median (IQR) | 24.1  (16.2-37.6) | 24.5  (19.5-36.2) | 25.4  (18.7-35.6) | 23.9  (19.0-36.7) | >0.9999 |
| Chi-square (male/female) and nonparametric ANOVA tests (age and body mass index [BMI]) on demographic and clinical parameters related to the different vaccination groups. Significant p-value is considered below 0.05; *p<0.05. | | | | | |

**Table S3.** Demographic characteristics of participants receiving a fourth COVID-19 vaccine dose.

| Vaccine schedule | | | | | | | | |  | | |  | | |
| --- | --- | --- | --- | --- | --- | --- | --- | --- | --- | --- | --- | --- | --- | --- |
| Primary | | **Booster 1** | **Booster 2** | |  | |  | | |  | | |  |  |
| All | Total | | | Total | | N. of individuals | | (% on total) | | | *n* = 20 (20.2%) | | |  |
|  |  |  |  |  |  | Gender | | Male | | | 8/20 (40.0%) | | |  |
|  |  |  |  |  |  | Age | | Years, median (IQR) | | | 59.0 (37.0-72.0) | | |  |
|  |  |  |  |  |  | BMI | | Median (IQR) | | | 24.5 (16.5-29.5) | | |  |
|  |  |  |  | mRNA-1273 | | N. of individuals | | (% on total) | | | *n* = 8 (8.1%) | | |  |
|  |  |  |  |  |  | Gender | | Male | | | 4/8 (50.0%) | | |  |
|  |  |  |  |  |  | Age | | Years, median (IQR) | | | 62.5 (52.0-72.0) | | |  |
|  |  |  |  |  |  | BMI | | Median (IQR) | | | 23.8 (19.5-26.0) | | |  |
|  |  |  |  | BNT | | N. of individuals | | (% on total) | | | *n* = 12 (12.1%) | | |  |
|  |  |  |  |  |  | Gender | | Male | | | 4/12 (33.3%) | | |  |
|  |  |  |  |  |  | Age | | Years, median (IQR) | | | 56.5 (37.0-69.0) | | |  |
|  |  |  |  |  |  | BMI | | Median (IQR) | | | 24.7 (16.5-29.4) | | |  |
|  | mRNA-1273 | | | Total | | N. of individuals | | (% on total) | | | *n* = 11 (11.1%) | | |  |
|  |  |  |  |  |  | Gender | | Male | | | 4/11 (36.4%) | | |  |
|  |  |  |  |  |  | Age | | Years, median (IQR) | | | 58.0 (46.0-68.0) | | |  |
|  |  |  |  |  |  | BMI | | Median (IQR) | | | 23.1 (16.5-25.6) | | |  |
|  |  |  |  | mRNA-1273 | | N. of individuals | | (% on total) | | | *n* = 7 (7.1%) | | |  |
|  |  |  |  |  |  | Gender | | Male | | | 3/7 (42.9%) | | |  |
|  |  |  |  |  |  | Age | | Years, median (IQR) | | | 62.0 (52.0-68.0) | | |  |
|  |  |  |  |  |  | BMI | | Median (IQR) | | | 23.1 (19.5-25.6) | | |  |
|  |  |  |  | BNT | | N. of individuals | | (% on total) | | | *n* = 4 (4.0%) | | |  |
|  |  |  |  |  |  | Gender | | Male | | | 1/4 (25.0%) | | |  |
|  |  |  |  |  |  | Age | | Years, median (IQR) | | | 55.5 (46.0-58.0) | | |  |
|  |  |  |  |  |  | BMI | | Median (IQR) | | | 20.9 (16.5-24.8) | | |  |
|  | BNT | | | Total | | N. of individuals | | (% on total) | | | *n* = 9 (9.1%) | | |  |
|  |  |  |  |  |  | Gender | | Male | | | 4/9 (44.4%) | | |  |
|  |  |  |  |  |  | Age | | Years, median (IQR) | | | 60.0 (37.0-72.0) | | |  |
|  |  |  |  |  |  | BMI | | Median (IQR) | | | 25.3 (20.5-29.4) | | |  |
|  |  |  |  | mRNA-1273 | | N. of individuals | | (% on total) | | | *n* = 1 (1.0%) | | |  |
|  |  |  |  |  |  | Gender | | Male | | | 1/1 (100.0%) | | |  |
|  |  |  |  |  |  | Age | | Years, median (IQR) | | | 72.0 | | |  |
|  |  |  |  |  |  | BMI | | Median (IQR) | | | 25.3 | | |  |
|  |  |  |  | BNT | | N. of individuals | | (% on total) | | | *n* = 8 (8.1%) | | |  |
|  |  |  |  |  |  | Gender | | Male | | | 3/8 (37.5%) | | |  |
|  |  |  |  |  |  | Age | | Years, median (IQR) | | | 58.0 (37.0-69.0) | | |  |
|  |  |  |  |  |  | BMI | | Median (IQR) | | | 25.5 (20.5-29.4) | | |  |
| ChAd/ChAd | mRNA-1273 | | | Total | | N. of individuals | | (% on total) | | | *n* = 10 (10.1%) | | |  |
|  |  |  |  |  |  | Gender | | Male | | | 3/10 (30.0%) | | |  |
|  |  |  |  |  |  | Age | | Years, median (IQR) | | | 59.0 (46.0-68.0) | | |  |
|  |  |  |  |  |  | BMI | | Median (IQR) | | | 23.3 (16.5-26.0) | | |  |
|  |  |  |  | mRNA-1273 | | N. of individuals | | (% on total) | | | *n* = 6 (6.1%) | | |  |
|  |  |  |  |  |  | Gender | | Male | | | 2/6 (33.3%) | | |  |
|  |  |  |  |  |  | Age | | Years, median (IQR) | | | 62.5 (52.0-68.0) | | |  |
|  |  |  |  |  |  | BMI | | Median (IQR) | | | 23.8 (19.5-26.0) | | |  |
|  |  |  |  | BNT | | N. of individuals | | (% on total) | | | *n* = 4 (4.0%) | | |  |
|  |  |  |  |  |  | Gender | | Male | | | 1/4 (25.0%) | | |  |
|  |  |  |  |  |  | Age | | Years, median (IQR) | | | 55.5 (46.0-58.0) | | |  |
|  |  |  |  |  |  | BMI | | Median (IQR) | | | 20.9 (16.5 -24.8) | | |  |
|  | BNT | | | Total | | N. of individuals | | (% on total) | | | *n* = 7 (7.1%) | | |  |
|  |  |  |  |  |  | Gender | | Male | | | 3/7 (42.9%) | | |  |
|  |  |  |  |  |  | Age | | Years, median (IQR) | | | 62.0 (37.0-72.0) | | |  |
|  |  |  |  |  |  | BMI | | Median (IQR) | | | 25.3 (20.5-29.4) | | |  |
|  |  |  |  | mRNA-1273 | | N. of individuals | | (% on total) | | | *n* = 1 (1.0%) | | |  |
|  |  |  |  |  |  | Gender | | Male | | | 1/1 (100.0%) | | |  |
|  |  |  |  |  |  | Age | | Years, median (IQR) | | | 72.0 | | |  |
|  |  |  |  |  |  | BMI | | Median (IQR) | | | 25.3 | | |  |
|  |  |  |  | BNT | | N. of individuals | | (% on total) | | | *n* = 6 (6.1%) | | |  |
|  |  |  |  |  |  | Gender | | Male | | | 2/6 (33.3%) | | |  |
|  |  |  |  |  |  | Age | | Years, median (IQR) | | | 61.0 (37.0-69.0) | | |  |
|  |  |  |  |  |  | BMI | | Median (IQR) | | | 25.2 (20.5-29.4) | | |  |
| ChAd/BNT | mRNA-1273 | | | Total | | N. of individuals | | (% on total) | | | *n* = 1 (1.0%) | | |  |
|  |  |  |  |  |  | Gender | | Male | | | 1/1 (100.0%) | | |  |
|  |  |  |  |  |  | Age | | Years, median (IQR) | | | 55.0 | | |  |
|  |  |  |  |  |  | BMI | | Median (IQR) | | | 20.2 | | |  |
|  |  |  |  | mRNA-1273 | | N. of individuals | | (% on total) | | | *n* = 1 (1.0%) | | |  |
|  |  |  |  |  |  | Gender | | Male | | | 1/1 (100.0%) | | |  |
|  |  |  |  |  |  | Age | | Years, median (IQR) | | | 55 | | |  |
|  |  |  |  |  |  | BMI | | Median (IQR) | | | 20.2 | | |  |
|  |  |  |  | BNT | | N. of individuals | | (% on total) | | | - | | |  |
|  |  |  |  |  |  | Gender | | Male | | | - | | |  |
|  |  |  |  |  |  | Age | | Years, median (IQR) | | | - | | |  |
|  |  |  |  |  |  | BMI | | Median (IQR) | | | - | | |  |
|  | BNT | | | Total | | N. of individuals | | (% on total) | | | *n* = 2 (2.0%) | | |  |
|  |  |  |  |  |  | Gender | | Male | | | 1/2 (50.0%) | | |  |
|  |  |  |  |  |  | Age | | Years, median (IQR) | | | 52.5 (49.0-56.0) | | |  |
|  |  |  |  |  |  | BMI | | Median (IQR) | | | 26.5 (25.2-27.8) | | |  |
|  |  |  |  | mRNA-1273 | | N. of individuals | | (% on total) | | | - | | |  |
|  |  |  |  |  |  | Gender | | Male | | | - | | |  |
|  |  |  |  |  |  | Age | | Years, median (IQR) | | | - | | |  |
|  |  |  |  |  |  | BMI | | Median (IQR) | | | - | | |  |
|  |  |  |  | BNT | | N. of individuals | | (% on total) | | | *n* = 2 (2.0%) | | |  |
|  |  |  |  |  |  | Gender | | Male | | | 1/2 (50.0%) | | |  |
|  |  |  |  |  |  | Age | | Years, median (IQR) | | | 52.5 (49.0-56.0) | | |  |
|  |  |  |  |  |  | BMI | | Median (IQR) | | | 26.5 (25.2-27.8) | | |  |

The number of subjects in each vaccination group, with relative percentages calculated based on the total at 21 month (i.e. 99 individuals) is reported alongside the median and IQR (25th-75th) values.

**Table S4.** Relationship between infected [N+] and uninfected [N-] subjects along the follow-up period of study.

| **Vaccine Schedule** | **Follow-up time points (Month)** | **p-value**  **N+ vs N-** |
| --- | --- | --- |
| ChAd/ChAd/mRNA-1273  vs  ChAd/ChAd/BNT | 9 | - |
|  | 12 | - |
|  | 21 | - |
| ChAd/ChAd/mRNA-1273  vs  ChAd/BNT/mRNA-1273 | 9 | - |
|  | 12 | *** |
|  | 21 | *** |
| ChAd/ChAd/mRNA-1273  vs  ChAd/BNT/BNT | 9 | - |
|  | 12 | - |
|  | 21 | - |
| ChAd/ChAd/BNT  vs  ChAd/BNT/mRNA-1273 | 9 | - |
|  | 12 | **** |
|  | 21 | * |
| ChAd/ChAd/BNT  vs  ChAd/BNT/BNT | 9 | * |
|  | 12 | - |
|  | 21 | - |
| ChAd/BNT/mRNA-1273  vs  ChAd/BNT/BNT | 9 | - |
|  | 12 | ** |
|  | 21 | ** |
| ChAd/ChAd/mRNA-1273  vs  ChAd/ChAd/BNT  vs  ChAd/BNT/mRNA-1273  vs  ChAd/BNT/BNT | 9 | - |
|  | 12 | **** |
|  | 21 | ** |
|  | 9-12-21 | **** |
| Intra-group and inter-group Chi-square (%N+ / %N-) related to the different vaccination groups at 9, 12 and 21 months after primary vaccination. Significant p-value is considered below 0.05 ( - = non-significant); *p<0.05, **p<0.01, ***p<0.001, ****p<0.0001. | | |

**Table S5.** SARS-CoV-2 anti-trimeric Spike IgG titers by vaccination schedule, time points and infection status.

| IgG titer (BAU/mL) |  | ChAd/ChAd/mRNA-1273 | | ChAd/ChAd/BNT | | ChAd/BNT/mRNA-1273 | | ChAd/BNT/BNT | |
| --- | --- | --- | --- | --- | --- | --- | --- | --- | --- |
|  |  | **N+** | **N-** | **N+** | **N-** | **N+** | **N-** | **N+** | **N-** |
|  | 9 months Median (IQR) | *n* = 26 (13.3%)  4940  (579-25600) | *n* = 85 (43.6%)  2040  (39.3-41400) | *n* = 4 (2.1%)  1940  (988-6160) | *n* = 19 (9.7%)  1944  (395-13160) | *n* = 3 (1.5%)  4200  (1094-17540) | *n* = 13 (6.7%)  3040  (711-4440) | *n* = 13 (6.7%)  6420  (725-40000) | *n* = 32 (16.4%)  1925  (605-22200) |
|  | 12 months Median (IQR) | *n* = 42 (24.4%)  2640  (123-17600) | *n* = 63 (36.6%)  1110  (39.2-10760) | *n* = 6 (3.5%)  1611  (628-2840) | *n* = 12 (7.0%)  1695  (102-7460) | *n* = 7 (4.1%)  2180  (670-10340) | *n* = 4 (2.3%)  1070  (961-1148) | *n* = 16 (9.3%)  4520  (237-9340) | *n* = 22 (12.8%)  1500  (287-20600) |
|  | 21 months Median (IQR) | *n* = 34 (34.3%)  2332.5  (333-9800) | *n* = 22 (22.2%)  2375  (367-15260) | n = 9 (9.1%)  2785  (920-12240) | *n* = 4 (4.0%)  1855  (117-2480) | *n* = 5 (5.1%)  4195  (1515-5750) | *n* = 1 (1.0%)  6050  - | *n* = 16 (16.2%)  3920  (1440-14500) | *n* = 8 (8.1%)  3280  (165-4555) |

Median and interquartile range (IQR) of SARS-CoV-2 anti-trimeric Spike IgG levels (BAU/mL) at 9, 12, and 21 months post-vaccination, stratified by vaccination schedule and infection status (N+ = infected, N- = uninfected). Data include sample size (n) and relative percentages of participants within each group at each time point. IgG levels are expressed as median values with IQR (25th-75th) in parentheses.

**Table S6.** SARS-CoV-2 anti-trimeric Spike IgG titers by primary vaccination schedule and time. points

| IgG titer (BAU/mL) | 9 months Median (IQR) | ChAd/ChAd | ChAd/BNT |
| --- | --- | --- | --- |
|  |  | *n* = 134 (68.7%)  2050  (39.3-41400) | *n* = 61 (31.3%)  2280  (605-40000) |
|  | 12 months Median (IQR) | *n* = 124 (71.7%)  1495  (39.2-17600) | *n* = 49 (28.3%)  1952  (237-20600) |
|  | 21 months Median (IQR) | *n* = 69 (69.7%)  2420  (117-15260) | *n* = 30 (30.3%)  3920  (165-14500) |
| Median and interquartile range [IQR] of SARS-CoV-2 anti-trimeric Spike IgG levels in ChAd/ChAd and ChAd/BNT immunized subjects at 9, 12, and 21 months post-primary vaccination. The number of subjects in each vaccination group, with relative percentages calculated based on the total at each time point (195 at 9 months, 173 at 12 months, and 99 at 21 months) is reported alongside the median and IQR (25th–75th) values. | | | |


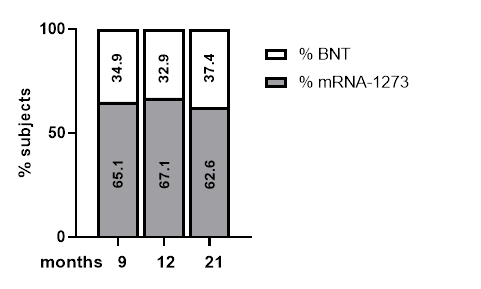


**Figure S1.** Percentages of subjects vaccinated with mRNA-1273 or BNT as third dose at month 9, 12 and 21.

**
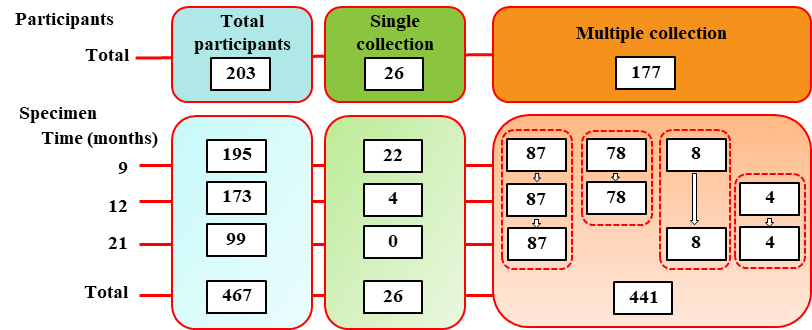
**

**Figure S2.** Flow diagram illustrating participant recruitment and specimen collection at each time-point and across multiple time points throughout the study.

**
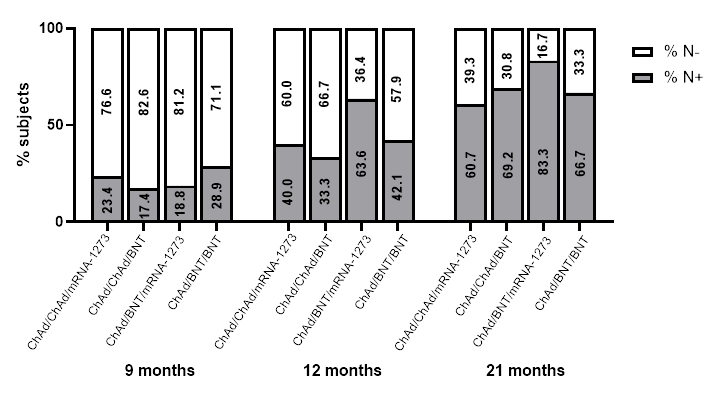
**

**Figure S3.** Percentages of SARS-CoV-2 infected/uninfected (i.e. N+ or N-, respectively) subjects vaccinated with four different administration schedules at 9, 12, and 21 months after vaccination.


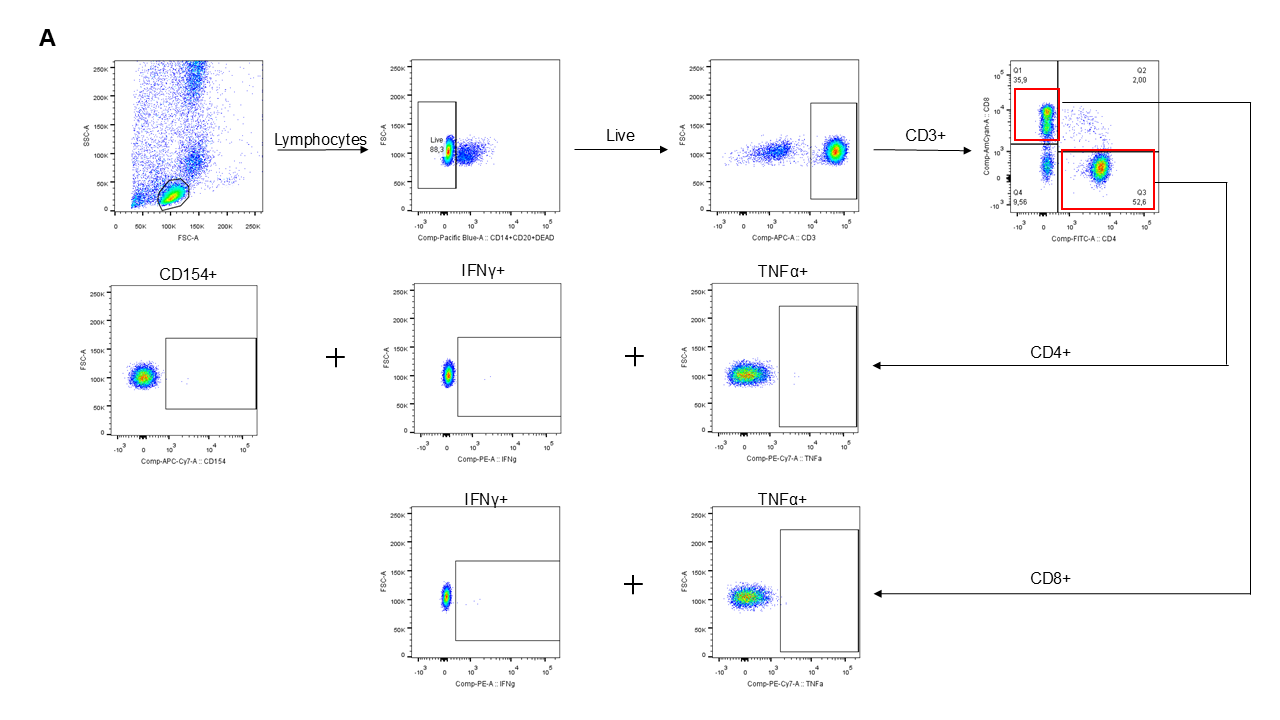

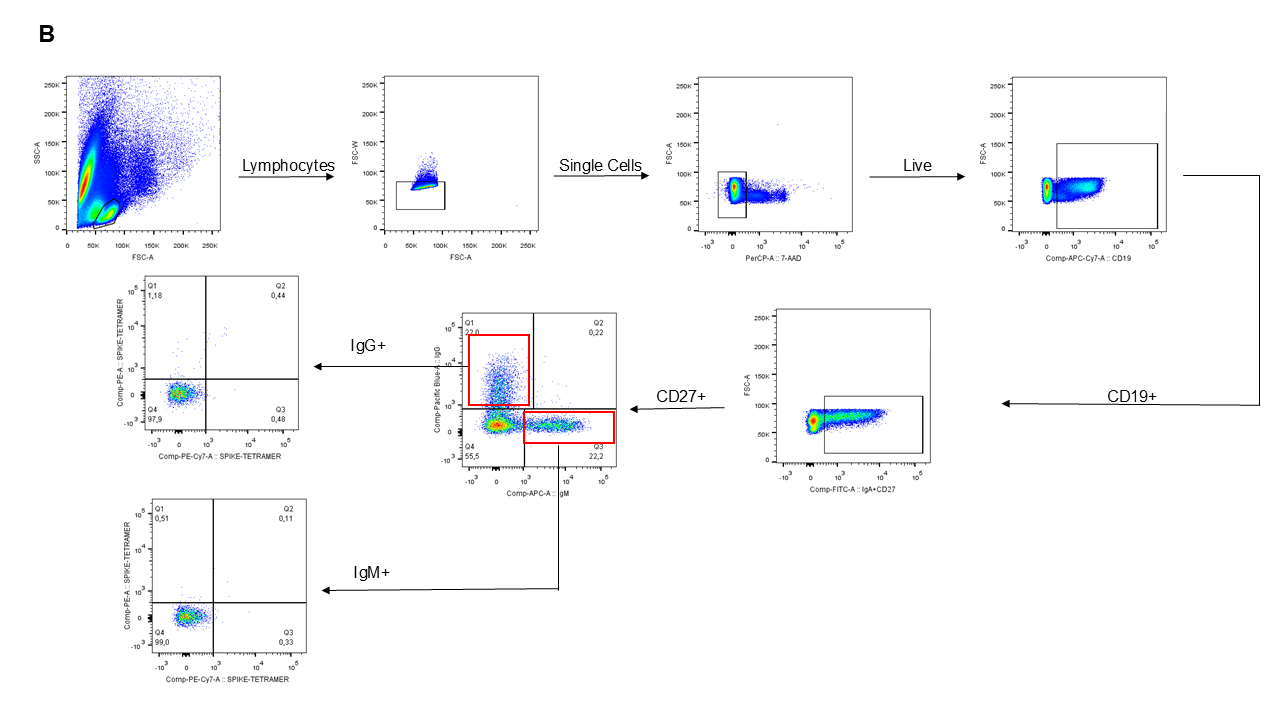


**Figure S4**. Representative plots of the gating strategy used to identify SARS-CoV-2 specific T cell and B cell populations. A) Gating strategy for quantifying intracellular cytokine expression in CD4+ or CD8+ T-cells; Lymphocyte gate > DEAD/CD14-/CD20- > CD3+ > CD4+ or CD8+ > mean fluorescence intensity [MFI] and percentage of TNF-α+, IFN-γ+, or CD154+ cells. Plots are shown as pseudocolor, arrows indicate sequential steps corresponding to the data in figure 4 and 5. B) Gating strategy to calculate the percentage of Spike specific MBC: Lymphocyte gate > Single cells > Live/Dead- > CD19+ > CD27+ > IgG+/IgM- or IgG-/IgM+ > Spike-Protein-PE+/PE-Vio® 770+. Plots are shown as pseudocolor, arrows indicate sequential steps corresponding to the data in figure 3.


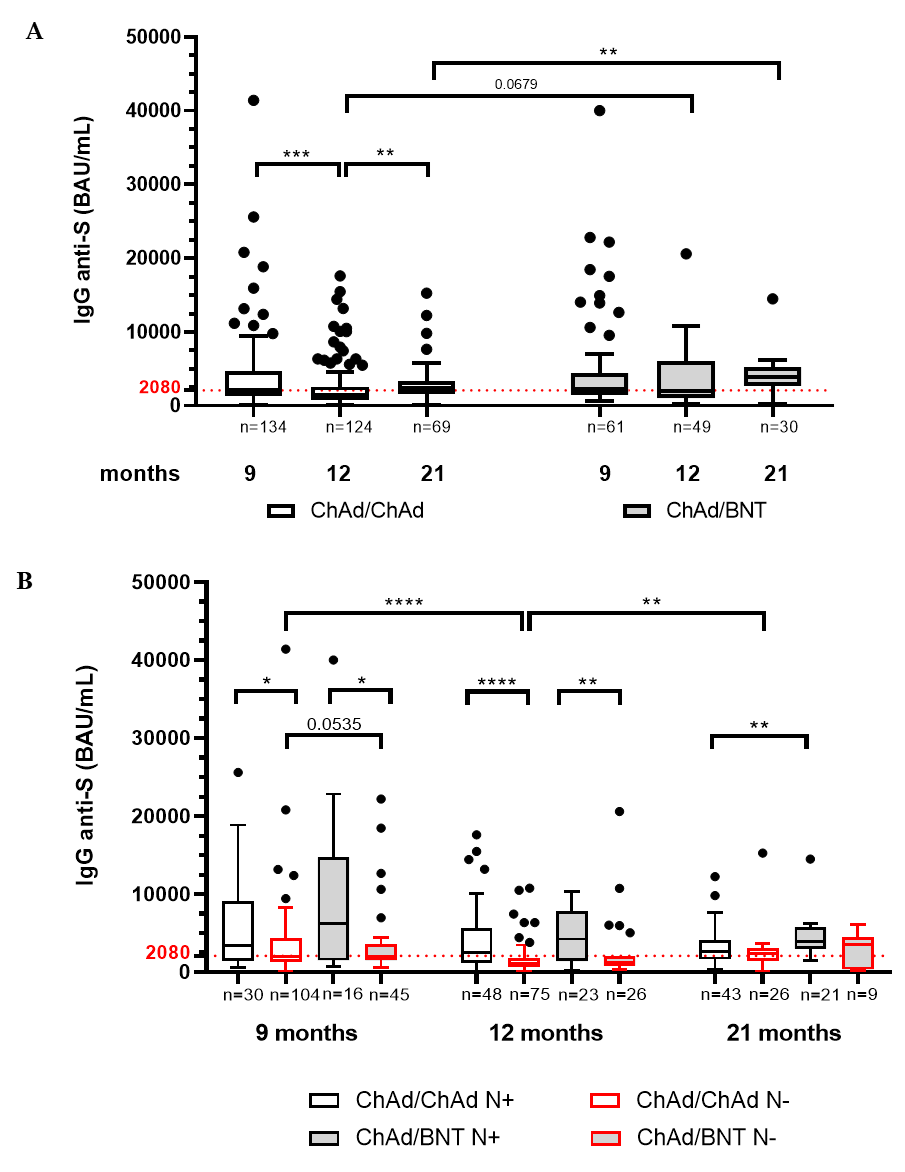


Figure S5. Inter-group comparison of SARS-CoV-2 anti-trimeric Spike protein IgG among ChAd/ChAd and ChAd/BNT vaccinated subjects at 9. 12 and 21 months post-primary vaccination. A) IgG levels in all subjects across the two vaccination schedules. B) IgG levels stratified by infection status (N+ = infected, N− = non-infected). Boxplots represent the median and interquartile range [IQR], with whiskers indicating the lowest and highest values according to Tukey-style plotting. The dotted red line denotes the upper limit of IgG anti-S quantification (2080 BAU/mL). Sample sizes for each group are reported below the x-axis. Kruskal Wallis test with Dunn’s post hoc multiple comparison and t test with Mann-Whitney post hoc pair-wise comparison. *p<0.05. **p < 0.01; ***P < 0.001; **** p < 0.0001.

**
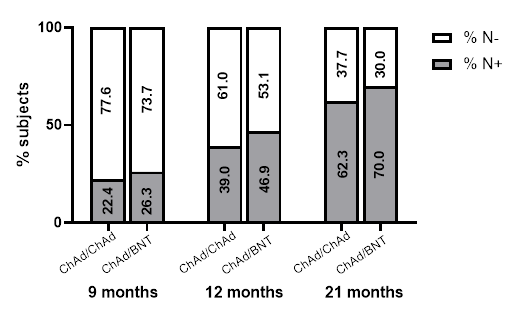
**

**Figure S6.** Percentages of SARS-CoV-2 infected/uninfected (i.e. N+ or N-, respectively) subjects vaccinated with ChAd/ChAd or ChAd/BNT schedule at 9, 12, 21 months after vaccination.


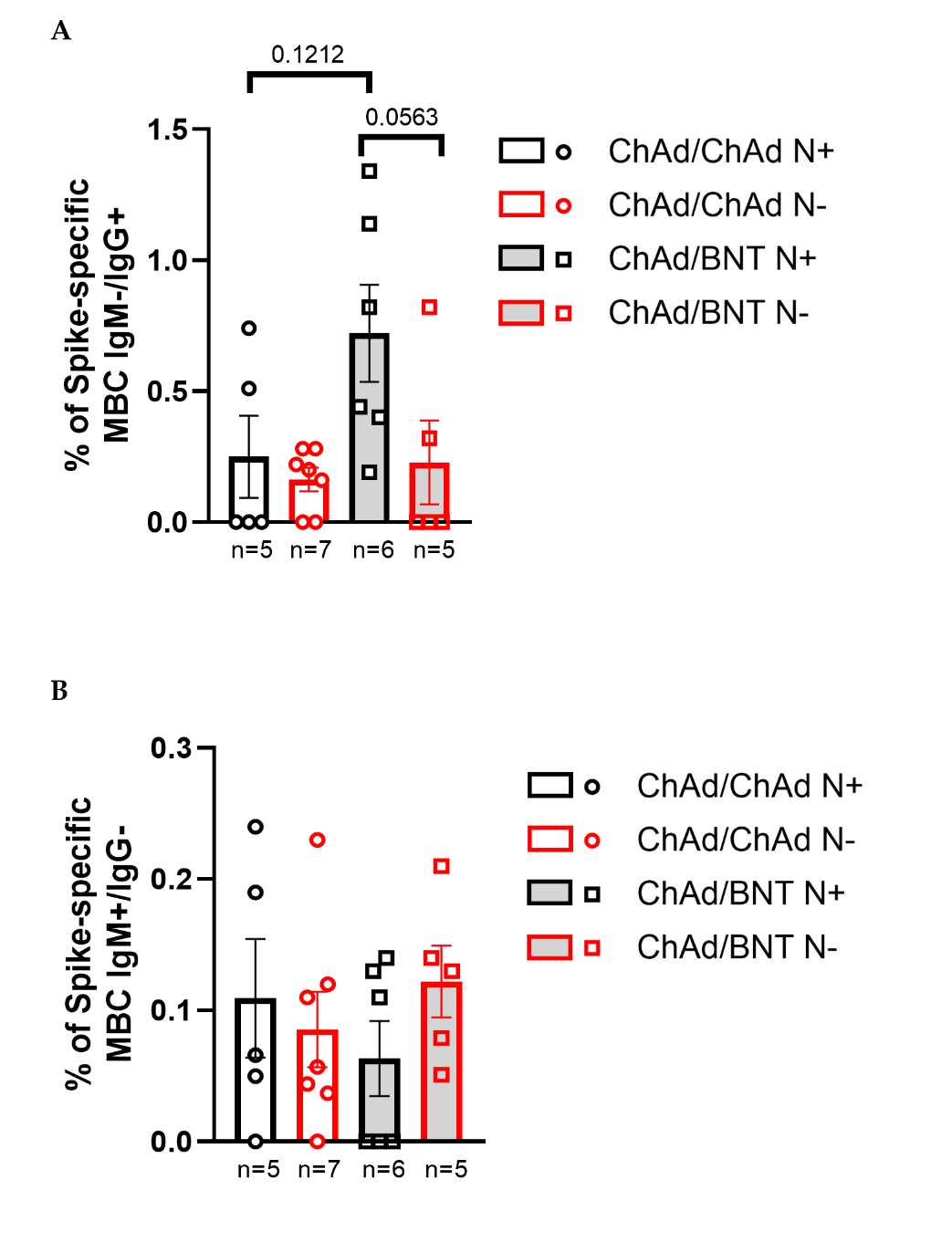


**Figure S7.** Inter-group comparison of the percentages of Spike specific memory B-cells [MBC] in SARS-CoV-2 infected/uninfected (i.e. N+ or N-, respectively) subjects vaccinated with ChAd/ChAd or ChAd/BNT schedule at 21 months after vaccination. A) Percentages of IgM- and IgG+ MBC. B) percentages of IgM+ and IgG- MBC. Bars represent the mean with standard error of the mean [SEM]. The number of subjects analyzed in each group is reported below the x-axis. Statistical significance was assessed using the Mann-Whitney U test.


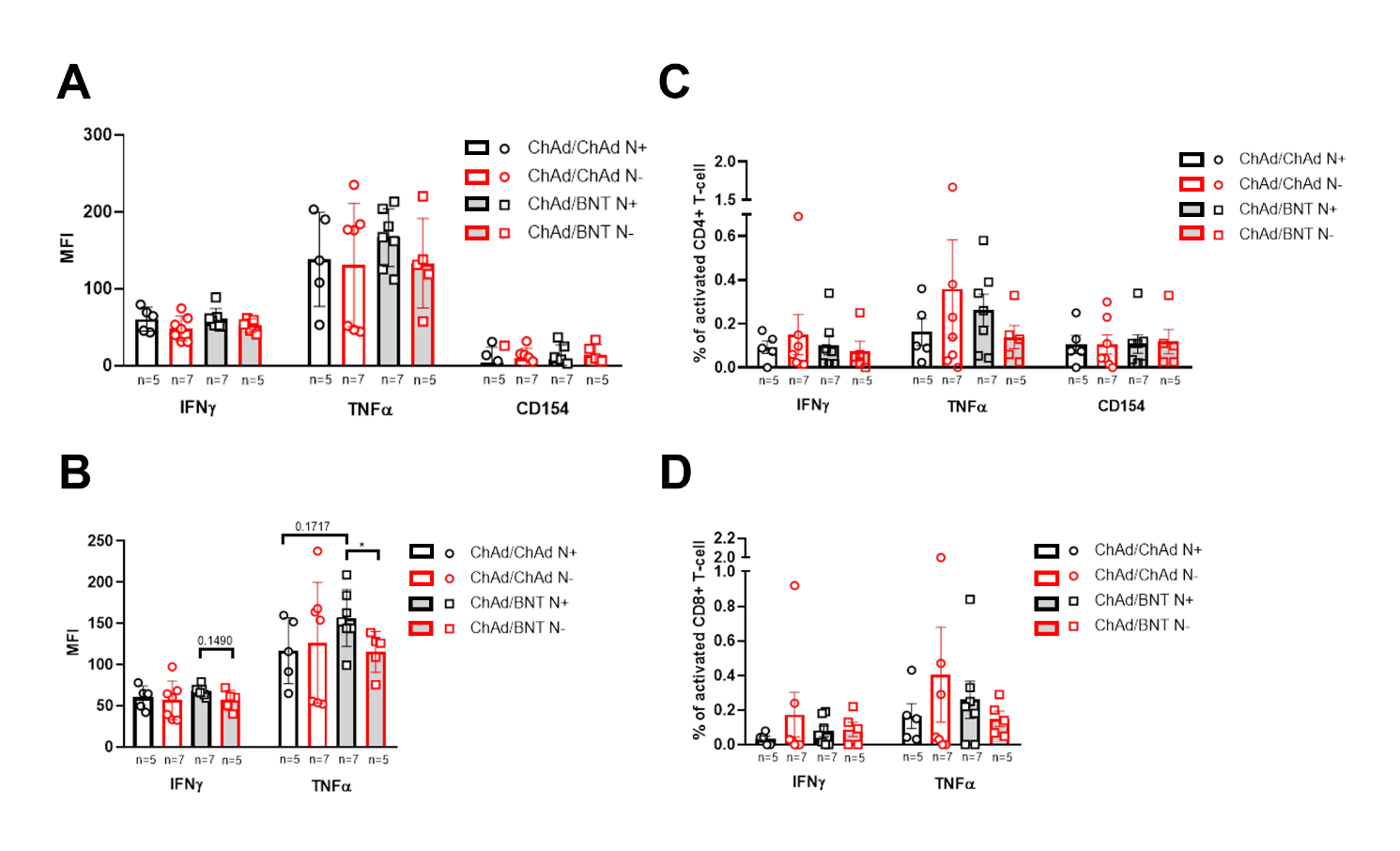


**Figure S8**. Inter-group comparison of intracellular cytokine expression in CD4+ and CD8+ T-cells in SARS-CoV-2 infected/uninfected (i.e. N+ or N-, respectively) subjects vaccinated with ChAd/ChAd or ChAd/BNT schedule at 21 months after vaccination. A) Mean fluorescence intensity [MFI] of IFNγ, TNFα, and CD154 expression in CD4+ T-cells. B) MFI of IFNγ and TNFα expression in CD8+ T-cells. C) Percentage of CD4+ T-cells expressing IFNγ, TNFα, or CD154. D) Percentage of CD8+ T-cells expressing IFNγ or TNFα. Bars represent the mean with standard error of the mean (SEM). The number of subjects analyzed in each group is reported below the x-axis. Statistical significance was assessed using the Mann-Whitney U test; *p < 0.05.


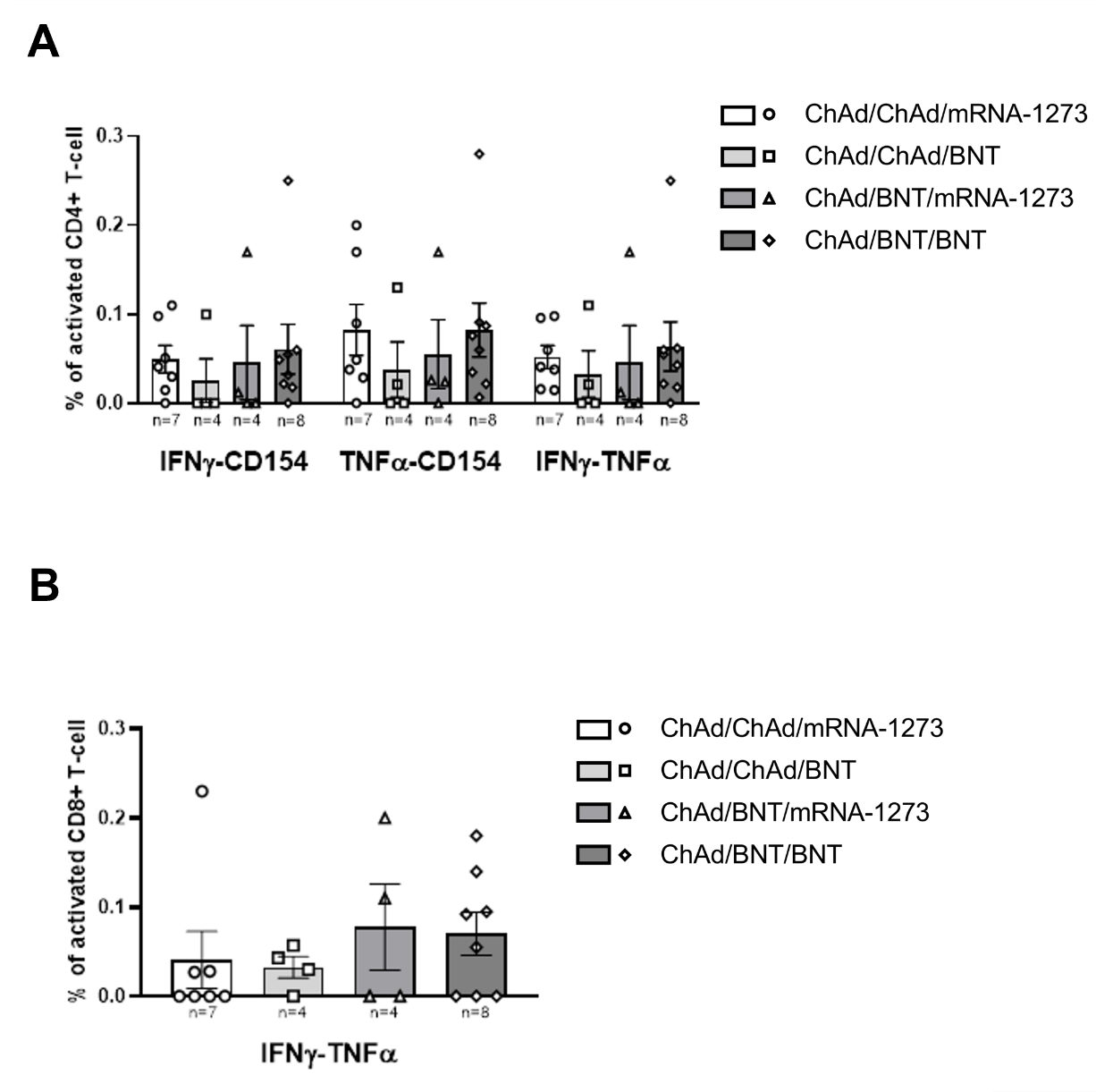


**Figure S9.** Inter-group comparison of the percentage of polyfunctional intracellular cytokine-expressing CD4+ or CD8+ T-cells among four vaccinated groups at 21 months post-vaccination. A) Percentage of CD4+ T-cells co-expressing IFNγ/CD154, TNFα/CD154 or IFNγ/TNFα. B) Percentage of CD8+ T-cells co-expressing IFNγ/TNFα. Bars represent the mean with standard error of the mean [SEM]. Sample sizes for each group are reported below the x-axis. Statistical significance was assessed using the Kruskal-Wallis test with Dunn’s post hoc multiple comparisons.


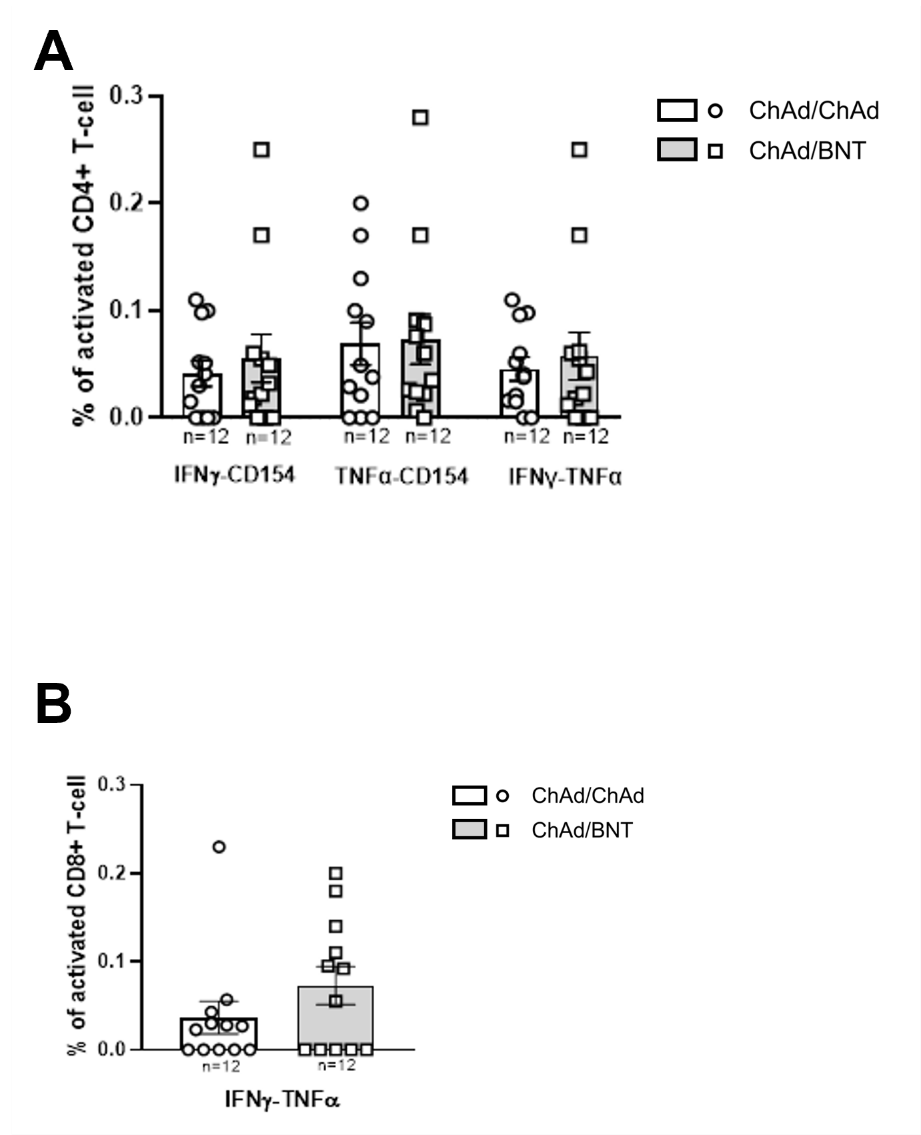


**Figure S10.** Inter-group comparison of the percentage of polyfunctional intracellular cytokine-expressing CD4+ or CD8+ T-cells between subjects receiving homologous (ChAd/ChAd) and heterologous (ChAd/BNT) vaccination schedules at 21 months post-vaccination. A) Percentage of CD4+ T-cells co-expressing IFNγ/CD154, TNFα/CD154 or IFNγ/TNFα. B) Percentage of CD8+ T-cells co-expressing IFNγ/TNFα. Bars represent the mean with standard error of the mean [SEM]. Sample sizes for each group are reported below the x-axis. Statistical significance was assessed using the Mann-Whitney U test.


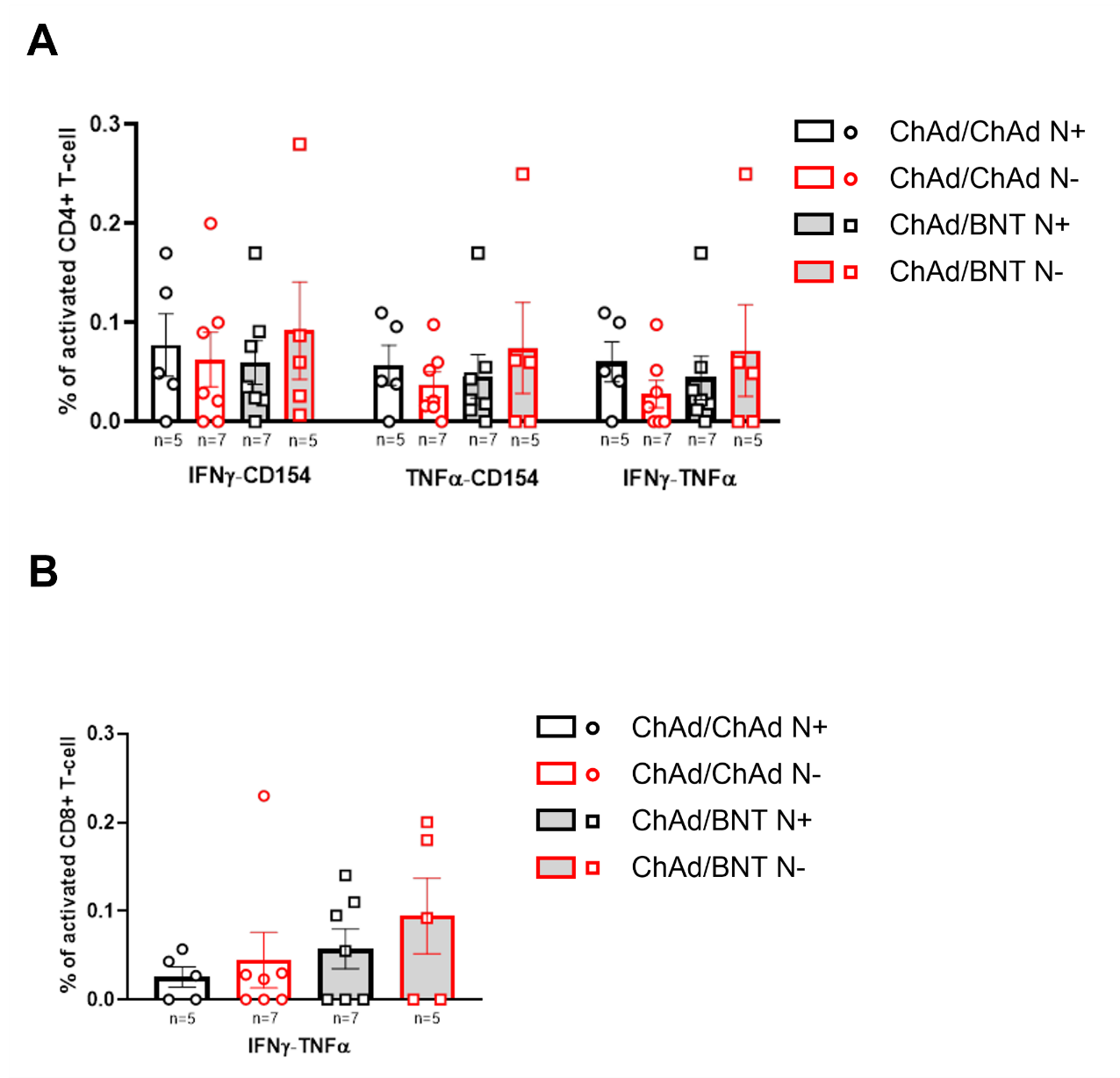


**Figure S11.** Inter-group comparison of the percentage of polyfunctional intracellular cytokine-expressing CD4+ or CD8+ T-cells in SARS-CoV-2 infected/uninfected (i.e. N+ or N-, respectively) subjects vaccinated with the ChAd/ChAd or ChAd/BNT schedule at 21 months after vaccination. A) Percentage of CD4+ T-cells co-expressing IFNγ/CD154, TNFα/CD154 or IFNγ/TNFα. B) Percentage of CD8+ T-cells co-expressing IFNγ/TNFα. Bars represent the mean with standard error of the mean (SEM). The number of subjects analyzed in each group is reported below the x-axis. Statistical significance was assessed using the Mann-Whitney U test; *p < 0.05.
